# Cutting-edge advancements in the synthesis, chemical structure, and applications of the lithium batteries and supercapacitors of metal–/covalent–organic frameworks

**DOI:** 10.1039/d6ra00378h

**Published:** 2026-03-27

**Authors:** Na Xiao, Yaqiong Wang, Kang Yang, Zhengjun Wang, Dian Zhao, Yulong Shi, Jufeng Qiao, Fang Qian

**Affiliations:** a Faculty of Engineering, Huanghe Science and Technology University Zhengzhou 450000 China angongyangkang@163.com; b School of Mechanical and Electrical Engineering, Linzhou College of Architectural Technology Anyang 456550 China; c School of Mechanical and Aviation Manufacturing Engineering, Anyang Institute of Technology Anyang 455000 China

## Abstract

Metal organic frameworks (MOFs) and covalent organic frameworks (COFs) are novel classes of advanced porous materials with high crystallinity, which have garnered significant research attention due to their distinctive structures and properties. However, the insufficient structural stability and poor conductivity are the major bottlenecks restricting their widespread application in new energy storage and conversion devices. A large number of studies have shown that by selecting appropriate synthesis methods and making targeted improvements, the crystal structure, pore characteristics and interface interactions of MOFs/COFs can be precisely controlled, thereby enhancing the bonding strength of covalent/coordination bonds, reducing the resistance of charge transfer, and improving their structural stability and conductivity. This paper describes the progress in MOF/COF research from the aspects of synthesis and chemical structure, summarizes the latest achievements, and presents their milestone applications in lithium-ion batteries and supercapacitors. Finally, it discusses the challenges and future prospects of developing high-performance MOF/COF energy storage materials.

## Abstract 2

The central part of the figure shows their typical structures. This review includes four sections that cover their synthesis methods, chemical structures, and applications in batteries and supercapacitors.

## Graphical abstract

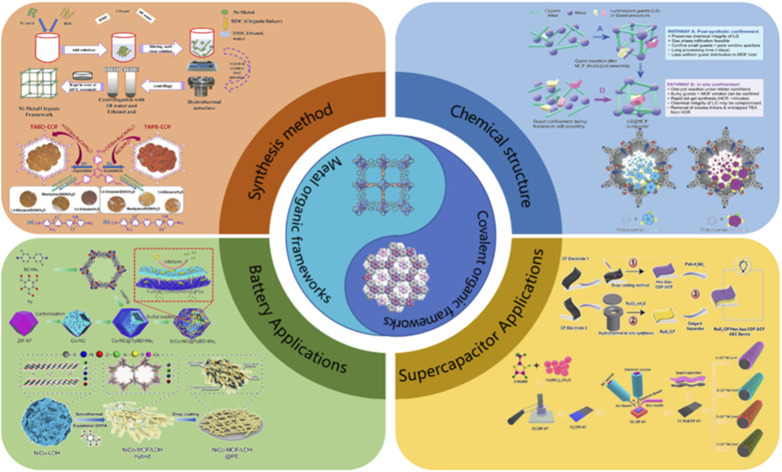

## 1. Introduction

The rapid development of society has brought convenience to the lives of people, but it has also led to many problems.^1^ The energy issue is among the most pressing concerns faced by humanity currently. Energy shortage has become an increasingly acute crisis, serving as a significant deterrent to global economic growth, and it could ultimately jeopardize human survival. Therefore, exploring and tapping new energy sources^2^ and reducing the energy consumption^3^ in equipment operation are the primary strategies to address the current energy crisis.

In recent years, porous materials have attracted considerable attention in various electronic fields, including batteries^4^ and supercapacitors (SCs),^5^ due to their unique properties. Among them, metal–organic frameworks (MOFs) and covalent organic frameworks (COFs) have shown particularly outstanding performance. MOFs are organic–inorganic hybrid materials that combine the characteristics of organic and inorganic materials,^6^ giving them a larger pore size range and specific surface area (SSA) than the common porous materials such as zeolites, aluminum phosphates, and activated carbon.^7^ COFs represent a new class of carbon-based porous nanomaterials characterized by their low density, high surface area, and tunable chemical composition.^8^ This enables researchers to create a variety of hybrid products by modifying their chemical composition and adjusting the covalent bonds.^9^ As a result, COFs offer considerable flexibility for designing complex products with diverse porosity.

The exceptional properties exhibited by MOFs/COFs have the potential to broaden their application across diverse scientific fields, particularly in the context of molecular catalysts for energy storage.^10,11^ Indeed, it is evident that both MOFs and COFs have been the focus of extensive research due to their considerable potential in energy storage systems. These materials can be utilized in a variety of ways, including their direct application as active catalytic materials.^12^ Alternatively, they can be used as precursors for the synthesis of porous carbon or metal oxides as active catalytic materials.^13^ For example, in a study by Xu *et al.*,^14^ novel 1D metal–organic nickel hydroxide nanorods were synthesized by the hydrothermal method and employed as electrode materials for SCs. Nickel-based MOF (Ni-MOF) nanorods exhibit exceptional uniformity regarding size, and they demonstrate high specific capacitance (mass-specific capacitance *C*_g_ and surface capacitance *C*_s_), remarkably high-rate capability, and outstanding cycle stability. After 1000 cycles at a current density of 1 A g^−1^, the capacitance remains at approximately 94.8%. The superior performance of the rod-like Ni-MOF is attributed to its large surface area, suitable pore size, active redox metal sites and rapid charge transport kinetics. In recent years, substantial research efforts have been dedicated toward the development of functionalized MOFs and COFs for diverse energy storage applications. The materials in question must satisfy several criteria including elevated SSA,^15,16^ considerable electrolyte chemical stability^17,18^ and adequate porosity.^19,20^ The emphasis on MOFs/COFs is mainly motivated by their characteristic porous structure, the benefits offered by the synthesis processes, and their potential applications.^21^ Consequently, there is a growing interest in exploring the potential of MOFs/COFs in energy-related applications.

Electrochemical energy storage systems are vital for advancing the global adoption of sustainable energy. Batteries and SCs are currently the most promising and environmentally friendly electrochemical energy storage systems, and they have been extensively studied in recent decades.^22,23^ Different types of batteries, including lithium batteries,^24^ zinc-air batteries,^25^ and nickel–cobalt batteries,^26^ have been broadly employed as energy storage systems for portable devices. However, alongside the growing trend towards global energy sustainability, the demand for efficient and reliable clean energy is increasing.^27^ The high energy density of batteries affords them the advantage of long-term discharge. Nevertheless, after long-term use, batteries inevitably suffer voltage and power loss, rendering them less suitable for meeting the demands of some high-power-density applications in the market. The advent of SCs offers a way to overcome these limitations, as they deliver higher power densities and can realize fast charging. However, the energy density of SCs is low, and there is still room for improvement.^28^ Electrode materials play a crucial role in determining the efficiency of electrochemical energy storage systems. Materials with high porosity, enhanced chemical stability, and numerous electrochemical active sites are considered advantageous for enhanced battery and SC applications.

**Fig. 1:**
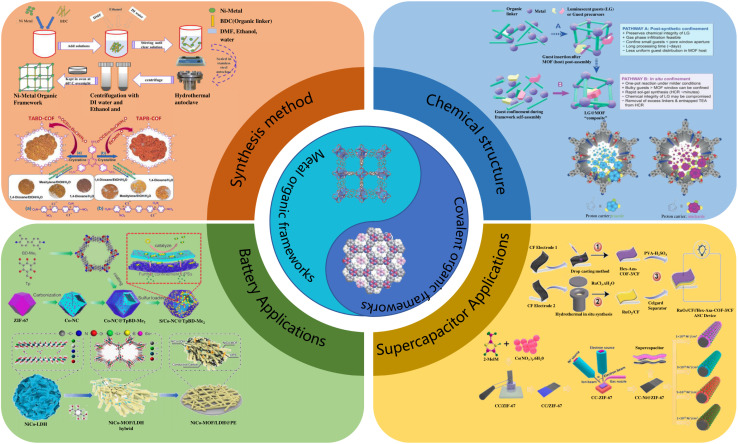
Schematic demonstrating the synthesis, chemical structure and applications of MOF/COF materials in batteries and SCs.^29–37^ Reproduced from ref. 29 with permission from Elsevier, copyright 2020. Reproduced from ref. 30 with permission from the American Chemical Society, copyright 2023. Reproduced from ref. 31 with permission from Elsevier, copyright 2024. Reproduced from ref. 32 with permission from the American Chemical Society, copyright 2022. Reproduced from ref. 33 with permission from Wiley, copyright 2018. Reproduced from ref. 34 and 36 with permission from Elsevier, copyright 2023. Reproduced from ref. 35 with permission from Elsevier, copyright 2022. Reproduced from ref. 37 with permission from MDPI, copyright 2022.

Motivated by the above considerations, this paper will focus on the synthesis, chemical structure, and functions of MOF/COF materials in battery and SC applications, which may contribute to the rapid development of related applications, as shown in Fig. 1. Synthesis methods are introduced in the second part, including solvothermal, microwave-assisted, mechanochemical, hydrothermal, and sonochemical methods. Subsequently, the chemical architecture of MOFs/COFs is examined, which serves as a guiding factor for their performance. Then, prominent topics concerning MOFs/COFs in batteries and the SCs are discussed. The operational mechanism of MOFs/COFs, with their characteristic high surface area, finely tunable porosity, and rich active sites, is also systematically analyzed. Finally, the principal challenges and future outlooks for MOF/COF materials are discussed.

## 2. Synthesis methods

MOFs and COFs constitute two novel categories of porous coordination polymers. MOFs are 3D materials composed of secondary building blocks formed from metal ions/clusters and organic ligands, while COFs are highly porous 2D or 3D organic solids composed of light elements (that is, H, B, C, N, and O). MOFs and COFs are highly conjugated scaffolds with modifiable electronic characteristics, elevated surface area, superior light and thermal stability, simple and comparatively low-cost preparation, and structural versatility. These attributes considerably extend their applicability.^38^ Recently, various synthetic methods have been explored to develop MOFs and COFs with more potentially advantageous characteristics. Several conventional synthesis methods are reviewed in this section and have proven successful in fabricating COFs/MOFs.^21^

The architecture and properties of MOFs/COFs are defined by the synthesis approach and conditions, such as temperature, reaction time, pressure, pH value, and solvent. Widely used synthetic strategies include solvothermal synthesis,^31,39^ microwave-assisted synthesis,^40,41^ sonochemical synthesis,^42,43^ mechanochemical methods,^44,45^ and hydrothermal synthesis,^46,47^ each of which has its own advantages and disadvantages. Table 1 summarizes the advantages and disadvantages (in terms of yield, time, cost, and crystallinity) of these five synthesis methods. Solvothermal synthesis offers high yield and is suitable for preparing materials with controllable morphology and pore structure, but it is time-consuming and costly. The other four methods, by contrast, involve lower costs. Microwave-assisted syntheses require extremely short times and produce materials with high crystallinity and uniform size, making the approach ideal for the rapid preparation of nanocrystals and small-batch, high-crystallinity samples. Mechanochemical syntheses, in contrast, offer very high yields, approaching 100%, but typically result in lower crystallinity; it is a fast process and ideal for solvent-free green synthesis. Hydrothermal syntheses offer high yields and superior crystallinity with uniform porosity, although it is time-consuming, making it appropriate for growing single crystals, large grains, and high-crystallinity materials. The approach is also suitable for large-scale and low-cost manufacturing. Ultrasonic syntheses require short reaction times and deliver the product with medium crystallinity and high yields; this approach is suitable for the preparation of room-temperature nanomaterials. Diverse targeted functionalities can be achieved through the selection of proper synthesis strategies and customized parameters.

**Table 1:** Comparison of the advantages and disadvantages (yield, time, cost, and crystallinity) of the five synthetic methods of MOFs/COFs

	Productivity	Time	Cost	Degree of crystallinity
Solvothermal synthesis	—	1–5 minutes	More than 50 US dollars per kilogram	Single crystal of 0.2 millimeters^48^
	More than 10 g	16 h	Costs are controllable	—^49^
Microwave-assisted synthesis	44% and 90%	25 s–4 h	Low cost	High crystallinity and uniform size^50^
	96.37%	15 min–1 h	Low cost	24.29% and uniform size^51^
Mechanochemical synthesis	∼100%	30–45 min	Low cost	High crystallinity^52^
	—	40 and 80 min	Low cost	Good crystallinity^53^
Hydrothermal synthesis	96%	24 h	Low cost	High crystallinity^54^
	84–96%	6 h	Low cost	High crystallinity and uniform porosity^55^
Sonochemical synthesis	—	∼20 min	Low cost	High crystallinity^56^
	60–98%	5 min–2 h	Low cost	High crystallinity^57^

### 2.1. Solvothermal synthesis

Solvothermal synthesis represents a systematic method for preparing MOFs and associated composites. This technology offers excellent production potential, operates at low temperatures, and requires minimal pressure.^58,59^ In the solvothermal method, it is very important to select the appropriate solvent for a reaction. Notably, solvents such as *N*,*N*-dimethylformamide (DMF),^60^ dimethyl sulfoxide (DMSO),^61^ toluene, and other organic solvents are commonly employed for the preparation of MOFs and MOF-COMs. Moreover, in addition to stimulating the development of new materials, these organic solvents can also be employed to direct the assembly of structures. DMF possesses an elevated boiling point and good solubility, and is the preferred organic solvent for the synthesis of MOFs and MOFs-COMs. In 2018, a metalloporphyrinic MOF (PCN-624) featuring perfluorophenylene functionalization was successfully prepared using DMF at 120 °C, with a reaction duration of 72 h.^62^ In 2019, Wu *et al.* employed the solvothermal method to improve the solubility of PCN-221(Fe_*x*_) in different concentrations of Fe.^63^ This resulted in the application of a photocatalyst designated MAPbI_3_@PCN-221(Fe_*x*_) for CO_2_ reduction. Ravipati *et al.* fabricated single-crystal Ni-MOF *via* a straightforward solvothermal method and precipitated it onto nickel foam (NF).^30^ The prepared Ni-MOF/NF electrode exhibited a specific current capacity of 8.8 A g^−1^ (compared with Hg/HgO) at an onset potential of 0.51 V. This was achieved because NF can transfer electrons quickly and has high conductivity on the surface between the electrode and the electrolyte. The porous architecture of the catalyst and the existence of a large number of metal charges generated by point defects enhance the electrocatalytic performance of the catalyst. Solvothermal synthesis of MOF and its composites with different length, width and morphology has also been confirmed in multiple studies, with polyhedral PCN-250-Fe_3_ crystals (with a diameter of about 13 mm),^30^ octahedral UiO-68 and UiO-68-TZDC (10–20 µm size range),^64^ octahedral Co-MOFs and Ni-MOFs mixture (∼200 nm size),^65^ new 2D Ni-MOF nanosheets (∼100 nm size),^66^ MIL-53(Fe) (∼50 mm size),^67^ and the octahedral MIL-100(Fe) (100–200 nm size range)^61^ being good examples.

Analogous techniques are also used to prepare COFs. Similar to the standard method for synthesizing inorganic zeolites in an autoclave, 3 to 7 days are required to prepare COF materials in a sealed heating container (100–130 °C) by the solvothermal method.^68^ The procedure involves placing the COF precursors in a heat-resistant glass tube, along with the necessary amount of solvent and, optionally, a catalyst. After degassing and sealing, the mixture is maintained at the desired temperature for a predetermined reaction time. Following this, the mixture is cooled to room temperature, and the product is collected by filtration, followed by drying at elevated temperatures (100–130 °C) to yield the COF powder. Solvothermal synthesis is the primary approach for generating new COFs. However, it is confronted with difficulties in controlling the shape and morphology. For instance, non-uniform solvent addition may lead to accelerated polymerization and uncontrolled phase separation. In response to these challenges, Su *et al.* proposed a two-step solvent feeding process for solvothermal molding of an imine-linked COF monomer with layered porosity.^69^ This study demonstrates that the two-step solvent feeding strategy can be effectively integrated with the traditional solvothermal synthesis to enhance the solution processability of COFs.

**Fig. 2:**
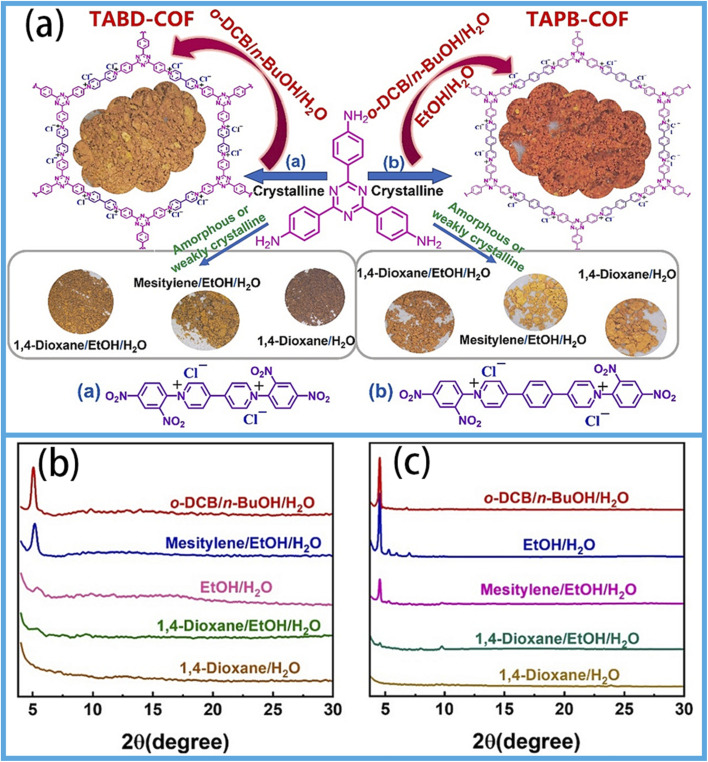
(a) Schematic of the synthesis of TABD-COFs and TAPB-COFs. PXRD patterns of TABD-COFs (b) and TAPB-COFs (c) obtained after treatment with different mixed solvents for 72 h; reproduced from ref. 31 with permission from Elsevier, copyright 2024.

In addition, the solvothermal method is also of great significance in industrial applications. Xu *et al.* synthesized two highly ordered ionic COFs (TABD-COFs and TAPB-COFs) by the solvothermal method; these COFs have many advantages, such as large adsorption capacity, good selectivity, good stability and clear pore structure.^31^ This makes it an exemplary material for extracting rare earths. Experimental investigations have substantiated that TABD-COFs and TAPB-COFs have highly ordered multilayer stacked crystal structures (Fig. 2a). Furthermore, their morphology and crystallinity can be tailored by changing the reaction conditions and the structure of the precursors. Unfortunately, TABD has an amorphous nature, as shown in the PXRD diagram in Fig. 2b, which makes the synthesis of TABD-COFs and TAPB-COFs difficult under the current synthetic parameters. Nevertheless, the successful synthesis of TAPB-COFs demonstrates that the structure of the building blocks plays an important role in the construction of TABD-COFs and TAPB-COFs. The crystallinity of the TAPB-COFs is corroborated by PXRD pattern analysis (Fig. 2c). Therefore, this study shows that the morphology and crystallinity can be modulated by adjusting the reaction conditions of the solvothermal method and by curating appropriate precursors, so as to engineer functional materials with excellent properties. Despite advantages such as mild reaction conditions and meticulous control over product dimensions, solvothermal synthesis is hindered by a series of challenges, including prolonged reaction durations, substantial consumption of organic solvents (such as DMF and DMSO) with inherent toxicity, susceptibility of product morphology and crystallinity to external factors, and significant environmental burdens. These drawbacks collectively exacerbate the temporal and environmental remediation costs and hinder large-scale production, and the inconsistent product quality can potentially compromise the reliability of practical applications in high-performance energy storage devices.

### 2.2. Microwave-assisted synthesis

Microwave heating has been employed in the domain of organic chemistry for several decades, particularly for the large-scale synthesis of COFs and MOFs. This method has been shown to significantly accelerate reaction kinetics and facilitate rapid crystal nucleation and growth.^70^ As early as 2006, Ni *et al.* utilized microwave-assisted heating technology to synthesize MOFs.^71^ The synthesis of MOFs, a process that previously required hours or days, can now be completed within a time frame of 30 seconds to 2 minutes. Nowadays, this synthesis method has demonstrated wide applicability, ranging from 30% to exceeding 90%. Furthermore, this method renders the growth process independent of the nucleation of walls or dust particles, thus enabling the synthesis of novel MOFs. Notably, the particle dimensions can be controlled by altering the precursor concentration. Moreover, Guo and his colleagues synthesized MOFs derived from rice-like Zn_2_GeO_4_ nanowire bundles by a microwave-assisted hydrothermal method.^72^ In comparison with the traditional hydrothermal/solvothermal technique, the microwave-heating method ensures uniform temperature across the entire reaction system. This method facilitates the preparation of a highly pure Zn_2_GeO_4_ phase, which is conducive to the synthesis of small-sized Zn_2_GeO_4_ crystals. This method is thus deemed to have substantial advantages over traditional hydrothermal/solvothermal methods.

In addition to the enhanced crystallinity and higher SSA, microwave synthesis has potential advantages including narrow particle size distribution, phase selectivity and easy morphology control.^21^ Wei and colleagues demonstrated a microwave-assisted synthesis of TpPa-COF with excellent crystallinity, larger surface area, and augmented CO_2_ adsorption capacity compared with solvothermal synthesis.^73^ Similarly, Alsudairy *et al.* synthesized a high-performance COF adsorbent to capture hazardous radioactive iodine, thereby overcoming the limitations of traditional solvothermal methods.^41^ As a feasible method to develop multicomponent COFs, microwave-assisted technology has the advantages of reduced reaction time and the ability to accurately customize the pore environment and adjust adsorption characteristics. It also has considerable prospects in environmental remediation and a range of niche applications. Microwave-assisted synthesis suffers from limitations including non-uniform heating at scale (causing product heterogeneity), stringent reactor material demands, precursor instability, and poor particle size control. These issues can be mitigated by designing multi-mode microwave systems for uniform power distribution, screening suitable precursors with the addition of stabilizers, and integrating template or surface modification strategies.

### 2.3. Mechanochemical synthesis

Whilst microwave and solvothermal reactions offer a series of MOF and COF materials with high crystallinity, stringent reaction conditions are requisite, including an inert atmosphere, a sealed reaction container, optimal temperature, and the use of a crystallization solvent. Consequently, there is a pressing need to explore more streamlined synthesis methods to facilitate rapid commercialization.

Mechanochemistry has great potential for the synthesis of high-value materials and is used in key reaction steps in many industries.^74^ The salient characteristic of this method is that it can be carried out in batch mode, mainly by using milling technology, or in continuous mode, for example, using screw extrusion.^75^ In addition, this method can be executed under solventless conditions, which is highly environmentally friendly.^76^ In synthetic chemistry, mechanochemistry has an extensive heritage. To date, it has been used in multicomponent (ternary and above) reactions to fabricate cocrystals with pharmaceutical activity, as well as in inorganic solid-state chemistry, polymer science, organic synthesis and numerous other domains. For example, in 2022, Negin Khosroshahi *et al.* prepared novel MOF-808 and NiFe_2_O_4_ nano-composites with various mass ratios through mechanochemistry and employed them for visible-light photocatalytic degradation of Meropenem and reduction of Cr(vi).^77^ In addition, Zhu *et al.* compared mechanochemically prepared (MOFs-5 (M)) and solvothermally (MOFs-5 (S)) prepared samples to develop efficient hydrogen storage media. The results demonstrate that across the pressure range of 0–10 MPa, the crystal structure inside MOFs-5 (M) is more regular and the distribution is more uniform, and the average size is only one-tenth that of MOFs-5 (S).^78^ These examples unequivocally underscore that the mechanochemical synthesis of MOFs has significant commercial potential. Importantly, mechanochemical methods have been widely used to synthesize various MOFs, including HKUST-1, ZIF-8, UIO-66 and MOFs-5, which have larger SSAs.

An analogous method is applied to the synthesis of COFs. Brown *et al.* demonstrated the liquid-assisted mechanochemical synthesis of COF adsorbents for efficient iodine capture.^79^ At room temperature, six types of imine-linked COFs with diverse pore sizes and functions were produced within one hour. Significantly, a representative COF exhibits exceptional crystallinity and a surface area of 1387 m^2^ g^−1^ within 1 minute of ball milling. The empirical results demonstrate that the mechanochemically synthesized COFs have an excellent iodine adsorption capacity, which is comparable with or higher than that of the solvothermally synthesized COFs and most reported COF adsorbent materials. In a similar vein, Hamzehpoor *et al.* reported an expeditious room-temperature mechanochemical synthesis of 2D and 3D boron-oxygen COFs.^45^ The COFs obtained in this study exhibit substantial porosity and crystallinity, with COFs-102 being the first example of a 3D COFs prepared by mechanochemistry; its surface area is approximately 2500 m^2^ g^−1^. In comparison with the solvothermal method, the mechanochemical method enabled a reduction in the solvent volume by approximately 20 times and the reaction time by about 100 times. Furthermore, the target COFs can be isolated in quantitative amounts without the need for additional treatment, aside from vacuum drying. Despite its environmentally benign and efficient nature, mechanochemical synthesis suffers from poor control of product structure, low crystallinity, pronounced equipment wear, and precursor degradation, although these can be mitigated through precise grinding control, liquid-assisted processing, and modular continuous manufacturing.

### 2.4. Hydrothermal synthesis

The hydrothermal synthesis strategy is regarded as exceptionally beneficial for the large-scale fabrication of MOFs/COFs owing to its simplicity, cost-effectiveness and environmental sustainability. The use of water instead of organic solvents as a reaction medium for the synthesis of MOFs/COFs is an appealing option. Liang *et al.* synthesized three new MOFs based on flexible tetradentate imidazole ligands L1 and L2 by the hydrothermal method.^47^ The results demonstrate that the three complexes exhibit enhanced thermal stability, with complexes 1 and 2 displaying robust solid-state fluorescence emission properties, indicating their potential as luminescent materials. Additionally, complex 1 exhibits an adsorption characteristic for C_60_ molecules. Similarly, Yang *et al.* used the hydrothermal method to synthesize two polyimide-linked COFs, namely HATN-PD-COFs and HATN-TAB-COFs.^46^ The constant-current intermittent titration technique and the theoretical calculation of density overtone collectively demonstrate that the HATN-PD-COFs has excellent high-rate performance (195 mA h g^−1^) at an ultrahigh current density of 10 000 mA g^−1^, and maintains a capacity retention of approximately 91% after 7000 cycles at 10 000 mA g^−1^. Moreover, Zhang *et al.* also used a hydrothermal synthesis to prepare COFs (ZVCOFs) with high crystallinity from predesigned zwitterionic building blocks.^80^ In this context, the addition of water not only substantially lowers the activation energy barrier for the reaction, thus enhancing the reversibility of the reaction but also promotes the hydration and orderly layered arrangement of ZVCOF, which, in turn, facilitates the crystallization. These examples undoubtedly demonstrate the important role of hydrothermal methods for the fabrication of high-quality MOFs/COFs. Hydrothermal synthesis, though simple and environmentally friendly, is constrained by narrow precursor applicability, insufficient ligand solubility, product agglomeration, and compromised structural stability, which can be ameliorated by adding co-solvents/surfactants, introducing templates, and developing low-pressure processes.

### 2.5. Sonochemical synthesis

The sonochemical approach is widely considered to be a highly efficient and environmentally benign synthesis route due to the fact that it is both rapid and environmentally friendly. In a recent study, Kaur *et al.* synthesized a 2D Zn(ii) and Cd(ii)-based organic framework (MOFs) by the sonochemical method.^81^ The effects of different irradiation times and precursor concentrations on the reproducibility of the morphology were studied. The outcomes of this study demonstrate that the synthesis of nanoparticles and spherical nanoparticles is feasible at low precursor concentrations, with the ultrasonic irradiation time playing a pivotal role in modulating the size of the resulting particles. These two kinds of MOFs have been used to selectively detect phenolic structural analogues, and have demonstrated efficacy. Similarly, Maleki *et al.*^82^ prepared water-stable MOFs/polymer composite-Tarbiat Modares University-10/poly pyrrole by a sonochemical method, and used it as an adsorbent to remove methyl red from polluted water, which achieved a good removal effect.

In 2012, Yang *et al.* for the first time obtained COFs-1 and COFs-5 crystals with excellent structural properties through ultrasonic chemical methods within 1 to 2 hours.^83^ This method offers a significantly reduced synthesis time (from 3 days to 1 hour), and yields smaller crystals (50–250 nm). Notably, their physical and chemical properties are comparable with, or even superior to, those of carbon nanotubes (CNTs) prepared by the vacuum solvothermal method. Similarly, the COF (TFPPy-AD) prepared by Wei *et al. via* the sonochemical method was characterized by the expediency of the approach, taking a mere hour to produce, and by the absence of any toxic reagents in its synthesis.^42^ The characterization results indicate that the TFPPy-AD possesses features such as substantial pore size, elevated crystallinity, and favorable thermal stability. Furthermore, COFs synthesized *via* ultrasonic chemistry demonstrate high adsorption capacity and selectivity, and rapid adsorption kinetics for flavonoids, indicating their potential for application in the separation and purification of flavonoids in natural medicines.

While the examples presented underscore the feasibility of synthesizing COFs and MOFs using green methodologies, it remains challenging to expand this approach to encompass a broader range of COFs and MOFs beyond the exemplars mentioned. To mitigate issues such as limited scalability, structural distortion, and narrow applicability, the use of high-power sonication systems, precise parameter tuning, and strategic precursor modifications can be considered.

## 3. Chemical structures

### 3.1. Structure and properties of MOFs

MOFs are novel crystalline porous materials assembled from metal ions and organic ligands. Their intrinsic chemical structure fundamentally dictates their performance. These core chemical structural features include secondary building units (SBUs, composed of metal ions or metal clusters, which determines the redox activity, coordination environment and structural stability of MOFs), organic ligands (which coordinate with SBUs through carboxyl and other functional groups to determine the pore size, specific surface area and topological structure of MOFs), and the pore chemical environment of the pores (which can regulate the polarity and hydrophilicity of the pores through ligand functionalization, thereby providing a chemical basis for selective adsorption, catalysis, *etc.*).^84,85^ The versatility of these chemical structure designs and the fine-tuning of pore structures make MOFs stand out as porous materials. Researchers can design and adjust the physicochemical attributes of MOFs from various organic building units, which remain unattainable in pure organic or inorganic systems.^32^ Chen *et al.* synthesized a hollow sea-urchin-shaped Ni-based MOF (NiPSC). Owing to the multi-center Ni-oxo SBU clusters and the large *d*-spacing distance, NiPSC demonstrated high specific capacity and excellent rate performance. The battery employing NiPSC as the positive electrode exhibited an 82.8% capacitance retention rate after 3000 cycles and achieved a maximum energy density of 28.81 W h kg^−1^ at a power of 425 W kg^−1^. This study demonstrates that incorporating multiple oxidation active sites and modulating the lattice spacing to tailor its internal structure can effectively improve the electrochemical performance of a material.^86^ Grafting various functional groups (−NH_2_, –NO_2_ and −Br) onto the carboxylic acid ligands of the sheet-like Ni-*p*-phenylene dicarboxylic acid type metal–organic framework nanosheets (Ni-BDC NAs) can be used to precisely engineer the microenvironment of Ni-BDC NAs while having minimal destructive effects on their structure and morphology. The overpotential of the functionalized materials is substantially mitigated, the long-term stability and structural stability are augmented, and the charge transfer resistance is attenuated. Among them, the performance of Ni-BDC-Br NAs is the optimal, with an overpotential of only 200 mV at a current density of 10 mA cm^−2^.^87^ In addition, compared with traditional porous carriers such as metal oxides, zeolites and carbon, MOFs have achieved the ability to expand the base surface without altering the base's topological structure through chemical modification. Therefore, MOFs can be compounded with other materials to construct excellent functional materials with improved overall performance.^88^

When MOFs are used as a functional material, to fully leverage their structural advantages and make up for the deficiencies of a single structure, their morphology can be regulated through composite strategies. Both the composite-forming method used and the spatial structure between the MOF and matrix material have a great impact on its performance.^89^ This section will introduce three typical composite-forming strategies: (1) MOFs embedded in nanopores, (2) MOFs fixed on the surface, and (3) MOFs coated into shells. In the first type, MOFs are embedded in nanopores. The stability of MOFs grown in nanospaces differs significantly from that of simple random mixtures. Nanopores effectively prevent the aggregation of MOFs. At the same time, porous materials increase the stability of the MOFs.^90^ For example, Farzaneh Mahmoudi *et al.* used the reflux method to confine the crystallization of MIL-68(Al) within the mesoporous channels of SBA-15, thereby forming the MIL-68(Al)@SBA-15 composite material. This structure embeds the MOF into the confined spaces of the mesoholes, significantly enhancing its water resistance. Compared with composite materials obtained through simple physical mixing, the MOFs that form through restricted crystallization can maintain their structural integrity even after repeated use, which indicates the protective and enhancing effect of the porous carriers on the stability of the MOFs.^91^

The second approach is to fix MOFs on the surface. For example, Du and his team designed a layered self-assembled MOF network to provide continuous ion transport and mechanical support for composite polymer electrolytes.^92^ This unique structure is achieved by constructing ordered MOF nanocrystals along the 1D polyimide fibers, providing continuous linear channels for lithium ions at the micron scale. The 1D MOF fibers are connected to form an integral 3D network for continuous transmission of Li^+^ in composite electrolytes. At the same time, the sub-nanopores and Lewis acid sites in the MOF nanocrystals can act as ion sieves to selectively restrict the movement of larger anions and promote the transport of Li^+^. In addition, the strong bonding between the MOFs and polyimide, coupled with the robustness of the polyimide skeleton, endows the MOF network with high mechanical strength and flexibility. Therefore, the synthesized composite electrolytes provide the high ionic conductivity and mechanical strength required.

The third kind of composite is MOFs coated into shells. The core–shell structures of MOFs and inorganic NPs (NP@MOF) are considered to be one of the easiest and most effective methods for achieving multifunctional applications of MOFs and inorganic NPs.^93^ The existence of a MOF shell structure not only greatly limits the aggregation and migration of NP nuclei, but also maintains their chemical stability. In addition, the MOF shell (offering structural adaptability, ordered crystal pores and multiple ligand sites) and NP core (with unique magnetic, electrical, optical and catalytic properties) can be effectively integrated.^94^ Owing to their unique structure and diverse nature, NP@MOF core shell nanomaterials have great potential in sensing^95^ and catalysis applications.^96^

### 3.2. Structure and properties of COFs

The synthesis and characterization of the COF materials have developed rapidly in the field of chemical science, owing to their distinctive structures and considerable potential in a range of application areas.^97,98^ A comprehensive understanding of these materials necessitates a detailed study of their structures. COFs are crystalline porous polymers which are formed from bottom to top from molecular building blocks with predesigned geometric shapes, linked *via* covalent bonds. They provide positional control of the building blocks in 2D and 3D space, thereby enabling the synthesis of rigid porous structures with high regularity while allowing fine-tuning of the chemical and physical properties of the network.^33^ Notably, the success of connecting 2D and 3D atomic systems to build extended frame structures shifts the chemistry of COFs from structure to method, highlighting the possibility of potential applications.^98^ Therefore, an in-depth study of the structure–performance relationship in COFs yields exceptional functional performance.

**Fig. 3:**
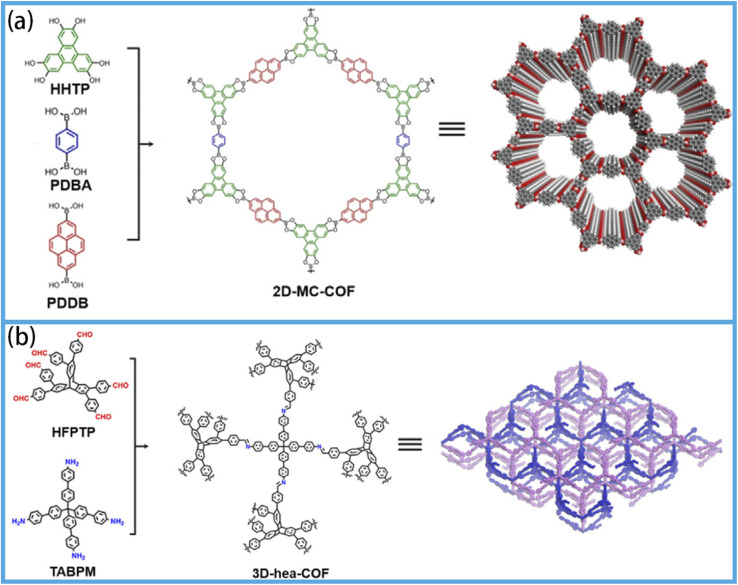
(a) Schematic of the construction of 2D-MC-COF, reproduced from ref. 100 with permission from the Nature Portfolio, copyright 2016. (b) Schematic of the synthesis of 3D-hea-COF, reproduced from ref. 101 with permission from the American Chemical Society, copyright 2021.

COFs are composed of multifunctional monomers bonded to one another *via* covalent bonds. In addition, the 2D structures are stacked layer-by-layer through weak interactions (such as hydrogen bonds and π–π stacking interactions). The result of this process is the formation of 1D channels. In recent years, the number of COF structures with different linkage types has exceeded 500, including borates, imines, triazines, hydrazones, azines and olefins.^99^ The preparation of 2D COFs typically involves the utilization of planar building blocks (Fig. 3a),^100^ while 3D COFs are formed *via* the condensation of 3D precursors (Fig. 3b).^101^

To date, the majority of COFs have been found to possess a 2D structure. The key challenges associated with the construction of 3D COFs lie in the scarce availability of 3D building units and notable crystallization issues.^102^ It is important to note that enhancing the clarity and diversification of COF structures offers significant advantages, particularly in the study of structure–performance correlations. This is of considerable significance for the exploration and development of new COFs for a range of functional applications.^16,103–105^

The flexibility and customization of the COF structure provide significant opportunities for the construction of functional COFs for a range of specific applications.^106–108^ In general, there are two approaches to adjusting the structures of COFs. The first approach is the bottom-up method, which prioritizes the design of monomers and directly synthesizes the target COFs.^109,110^ Reacting hexaazatriphenylene (HATp), as a rigid conjugated monomer, with aromatic diamine/dialdehyde monomers, forms conjugated networks of COFs with an ordered channel. The planar conjugated structure of the HATp monomer is linked by strong covalent bonds, making the MOF framework highly chemically stable and structurally rigid, with a longitudinal contraction rate of the pore channel of less than 2%. The high negative charge of the HATp framework enhances the affinity for metal ions (Li^+^, Na^+^, K^+^, and Zn^2+^), forming stable ligands with metal ions, and inhibits ion aggregation and shuttling, and the rigid conjugated structure provides a continuous electron transmission path. Moreover, the ordered pore channels shorten the ion migration distance, which can improve the electron conduction efficiency. As a lithium-ion electrode material, the specific capacity reaches 280 mA h g^−1^ at a current density of 0.1 A g^−1^.^111^ The alternative approach involves post-synthesis modification.^112^ This approach involves the introduction of new functional groups into the original COFs, with the objective of adjusting their surface or pore environment while maintaining the integrity of the original COF structure. After synthesis and modification, the potential application scope of the COF materials expands significantly. For instance, TTBT-COF was functionalized with triazine rings to be used as a separator for lithium–sulfur batteries. The highly conjugated and electronegative characteristics of the triazine rings endow COFs with strong polarity and electron transport capability. Moreover, while the high electronegativity of the triazine rings exerts electrostatic repulsion, preventing the migration of polysulfide anions, the conjugated structure enhances electron conduction and accelerates the kinetics of polysulfide redox reactions. The initial capacity at 1C current reaches 1022 mA h g^−1^, and the average capacity decay rate per cycle is 0.057%.^113^

In a similar manner, the modification and practical application of COFs are influenced by their stability. In general, COFs exhibit favorable thermal stability and robust covalent bonds, with the capacity to preserve structural integrity at temperatures ranging from 250 °C to 450 °C under an inert gas.^99^ Despite the challenges related to the chemical stability of COFs, significant advancements have been made in developing methodologies aimed at enhancing their structural stability.^114,115^ It is important to note that COFs exhibit highly ordered, versatile and permanent porosity, which renders them highly promising for a variety of applications.^116^ Furthermore, in addition to the pore size, the pore environment of the COFs can be adjusted with a high degree of accuracy.

Consequently, the structure of COFs plays an important role in their application in many fields. The advantages of such structural analyses include the following: (1) defining the structural characteristics of COFs can provide a theoretical basis for synthesizing COFs with specific functions. (2) Understanding the relationship between the structure and properties of COFs is helpful for predicting the properties of materials and for making targeted modifications. (3) In energy storage devices, such as batteries and SCs, exploring the COF structure can help guide the design of electrode materials with high ionic conductivity, good stability and high specific capacity. In addition, exploring their structure is helpful for discovering and exploring new functions and application scenarios of COFs in the field of energy storage.

## 4. Application of energy storage batteries

Confronted with the dual challenges of the energy crisis and environmental protection, exploring novel solutions has become imperative. The growing prevalence of electric vehicles has led to a shift in the automotive landscape, with traditional fossil fuel vehicles gradually being replaced by their electric counterparts.^117^ In addition, batteries are widely explored as reliable electrochemical energy storage devices. However, current battery designs have defects such as low power density and short service life.^118,119^ Crystalline porous materials have attracted great interest in the field of energy storage, especially in the battery field, because orderly porous frameworks can provide fast ion transmission and storage paths without large volume changes.^29^ Consequently, MOFs and COFs stand out from the wide range of functional materials, and they show great potential for battery applications.

### 4.1. Application of MOFs in energy storage batteries

MOFs are hybrid materials composed of different metal nodes and organic linkers, featuring ultra-high porosity, large SSA, and easy functionalization. They can provide superior electronic transfer and rapid mass transfer in various industrial applications, and have excellent electrochemical properties.^120^ These characteristics make them promising candidates for developing lithium-based batteries (such as lithium-ion batteries, lithium sulfur (Li–S) batteries, and lithium oxygen batteries (LOB)) with excellent electrochemical properties.^121^

#### 4.1.1 Lithium-ion batteries

The ultra-high porosity, multi-functionality, structural diversity and tunable chemical composition of MOFs offer great potential for their deployment as advanced electrode materials in rechargeable batteries.^122^ Lithium-ion batteries exhibit excellent electrical performance and safety profiles, and they have good cycle stability; thus, they are one of the most promising energy storage components in power equipment^123,124^ and can make a significant contribution to achieving “carbon neutrality”.^125^

Lithium-ion batteries consist of an anode, a cathode, an electrolyte, a diaphragm and a shell.^126^ In general, the working principle of lithium-ion batteries is that the cathode and anode exchange lithium ions in the electrolyte to store or release electrical energy. The working efficiency and capacity of a battery mainly depend on the cathode, anode and electrolyte.^127^ The separator and electrolyte ensure the normal operation of the battery, while the positive and negative electrode materials determine the battery capacity.^128^ The electrode materials serve as the primary factors governing the electrochemical performance of lithium-ion batteries. The inherent ultra-high porosity and large SSA of MOFs are conducive to the penetration of electrolytes and can effectively withstand the volume expansion that occurs during the storage of lithium ions. In addition, their designable components enable the incorporation of electroactive sites, laying the foundation for the development of candidate electrode materials suitable for lithium-ion batteries.^129^ Lithium-ion batteries achieve energy storage and release through the reversible insertion and extraction of lithium ions between the positive and negative electrodes. Taking the typical positive electrode material LiCoO_2_ and the negative electrode material graphite as examples, the basic electrochemical reaction can be expressed as follows:

Cathode reaction:

1 LiCoO_2_ ⇌ Li_1−*x*_CoO_2_ + *x*Li^+^ +*x*e^−^

and anode reaction:

2 C_6_ + *x*Li^+^ + *x*e^−^ ⇌ Li_*x*_C_6_

The formation of the solid electrolyte interphase (SEI) on the anode during initial cycles can be expressed as:

3 2Li^+^ + EC/DMC + 2e^−^ → Li_2_CO_3_ + organic compounds.

The high porosity and controllable structure of MOFs can optimize the above electrochemical processes in multiple aspects: promoting the kinetics of lithium ion transport, adapting to the structural deformation during the cycling process, and potentially enhancing the intrinsic activity of the electrode materials through chemical modification. Therefore, MOFs and their derivatives have become important candidate systems for electrode materials in lithium-ion batteries. Table 2 shows the performance of MOF hybrid electrode materials in lithium-ion batteries developed in recent years.

**Table 2:** Electrochemical properties of MOFs and their derivatives as the electrode materials for lithium-ion batteries

Materials	Current density (mA g^−1^)	Specific capacity (mA h g^−1^)	Cycles
MOF-SnO_2_ NP	—	1050.8	100 (ref. 130)
Si/CoMo@NCP	500	1013	100 (ref. 131)
Co-MOF 2	100	732	200 (ref. 132)
Fe_2_O_3_-Co_3_O_4_/NPC	2000	406	1000 (ref. 133)
PbSe/SC	100	717	100 (ref. 134)
Fe-HHTP	355	1142	220 (ref. 135)
ZIF-67@rGO	2000	1002	2500 (ref. 136)
NiMnCo-MOF	15	685	50 (ref. 137)
Co-ZnO/C and Co-Co_3_O_4_/C	37.2	898 and 784	100 (ref. 138)
FNO@NCNFs	1000	748.5	900 (ref. 139)
Fe-Tp	1000	743	500 (ref. 140)
Cu-IM/Co-MOF 250 and Cu-IM/Co-MOF	50	834 and 54.3	75 (ref. 141)
Co-CAT	200	404	100 (ref. 142)

At present, the negative electrode material used in most lithium-ion batteries on the market is graphite. However, the theoretical gravimetric capacity of conventional graphite anodes is 372 mA h g^−1^.^143^ This value cannot meet the power density and reliability requirements required for large-scale applications. Therefore, developing new types of batteries with high capacity, low charging potential and low production cost is an important and urgent task.^128^ Owing to the ultra-high SSA of the catcher contacts, abundant lithium-ion storage active sites, and tunable pores that enable lithium-ion migration, MOFs are considered one of the most promising candidates to replace graphite anodes in current lithium-ion batteries.^144^ In experiments, the polycrystalline MOF-177 has been employed as an anode material for lithium-ion batteries, providing a high initial irreversible capacity of 400 mA h g^−1^, which is a favorable performance. However, after only two cycles, the capacity attenuates rapidly to 105 mA h g^−1^.^145^

**Fig. 4:**
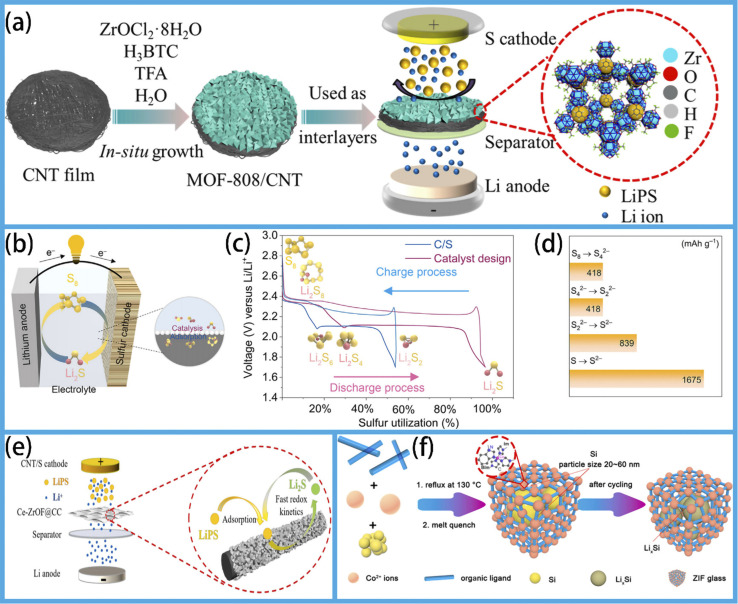
(a) Schematic of the synthesis of MOF-808/CNT interlayers, reproduced from ref. 148 with permission from Elsevier, copyright 2022. (b) Oxidation and restoration reactions occurring in Li–S batteries. (c) Voltage distribution at the carbon/sulfur (C/S) cathode. (d) The theoretical discharge capacity of each stage in a battery, reproduced from ref. 149 with permission from KeAi Communications Co., Ltd, copyright 2023. (e) Ce-ZrOF@CC function diagram of the middle layer, reproduced from ref. 150 with permission from Elsevier, copyright 2022. (f) Synthesis of SiZGC materials and the protective effect of the ZIF glass matrix, reproduced from ref. 144 with permission from Elsevier, copyright 2022.

Long-life battery materials and battery design have always been the goals of research. However, due to their electrochemical properties, lithium-ion batteries are affected by the battery design (battery structure, electrode engineering, *etc.*), chemical composition (active materials, electrolytes, additives, *etc.*), mode of use, and operating conditions, and will experience irreversible ageing.^146^ Yan *et al.* obtained Si@calbolt-ZIF-62-glass composites (SiZGC) by *in situ* growth of a MOF on the Si surface, followed by melting and quenching the MOF to a glassy state. The preparation is shown in Fig. 4f. Studies show that the disordered network structure of ZIF glass can provide additional channels for the diffusion and storage of Li^+^, thereby effectively enhancing the electrochemical performance of the anode in lithium-ion batteries. Additionally, the ZIF glass phase can mitigate volume variations and inhibit the agglomeration of silicon nanoparticles during the lithiation/delithiation process. After 500 cycles of the 10SiZGC-based anode, the specific capacity at a current density of 1 A g^−1^ is 650 mA h g^−1^.^144^ Furthermore, the metal–organic framework (MOFs-199, namely, Cu_3_(BTC)_2_) is deposited on the surface of graphene oxide (GO) in a layer-by-layer (LBL) coating approach to fabricate the Cu_3_(BTC)_2_@GO composite anode material. Owing to the porous nature of the MOFs, at a current density of 100 mA g^−1^, the composite delivers an initial charge/discharge capacity that reaches 1200/1420 mA h g^−1^, with a coulombic efficiency of 85%. After 100 cycles, the reversible discharge capacity is 1296 mA h g^−1^, the coulombic efficiency is 98.9%, and the capacity retention rate is 91%. When the current density increases from 100 mA g^−1^ to 2000 mA g^−1^, its specific capacity decreases from 1352 mA h g^−1^ to 836 mA g^−1^. When it is restored to 100 mA g^−1^, the capacity recovery rate reaches 94%.^147^

Although MOFs and their derivatives possess many advantages, they face a variety of critical challenges as electrode materials, which hinder their practical implementation. Low driven rate, poor cycling stability, and electrochemical instability constitute the primary obstacles for the practical application of MOF electrodes.^151^

#### 4.1.2 Lithium–sulfur batteries

In comparison with lithium-ion batteries, Li–S batteries exhibit high theoretical rate capacity (1675 mA h g^−1^) and theoretical energy density (2600 W h kg^−1^), with the latter being nearly five times that of traditional lithium-ion batteries.^152–154^ In addition, as the cathode material for Li–S batteries, sulfur (S) is naturally abundant, low in cost, and environmentally friendly, laying a solid foundation for its large-scale application.^155^ However, Li–S batteries still suffer from critical unresolved challenges, such as low active-material utilization, poor cycling stability, the polysulfide shuttle effect, and sluggish reaction kinetics.^26^ In recent years, various strategies to improve the working performance of Li–S batteries have been proposed in view of the excellent performance of MOFs, including developing new cathode bodies and electrolytes, modifying separators and anodes, and inserting intermediate layers.^156–160^ For instance, when sulfur is embedded in the pores of carbonized ZIF-8 as the cathode host, the ultrahigh micropore density inhibits the dissolution and shuttling of polysulfides. After 100 cycles, a reversible capacity of around 490 mA h g^−1^ can be achieved based on sulfur mass, and it also exhibits stable cycling performance and high coulombic efficiency.^161^ Of these four approaches, inserting an intermediate MOF-based layer between the cathode and the separator has been proven to be a simple and effective method.^34,162,163^

Zirconium-based MOFs (Zr-MOFs), such as MOFs-808, exhibit several key advantages, such as excellent hydrophilicity, high chemical stability and relatively large pore sizes, which are suitable for use as membrane-based interlayer materials.^164^ Wang *et al.* successfully fabricated a continuous Zr-based MOFs-808 film (MOFs-808/CNT) *via* a CNT thin-film substrate.^148^ The design of the MOFs-808/CNT interlayer is shown in Fig. 4a. A dense MOFs-808 film is prepared on a CNT film *via* an *in situ* hydrothermal method and employed as an ion-selective interlayer to inhibit the shuttle effect in Li–S batteries, effectively blocking the polysulfides while enabling rapid transport of lithium ions, providing long-lasting cycle stability for Li–S batteries. In addition, the excellent electrical conductivity of CNT films can remedy the poor electrical conductivity of MOFs-808 films, giving a rate capacity of 707.3 mA h g^−1^ at 5 °C. Zhou *et al.* impregnated Zr-based MOFs-808 films with cerium (Ce) salts and carbonized them to fabricate ZrOF/carbon composites based on carbon fiber cloth (Ce-ZrOF@CC).^150^ Synergistic effects between the materials reduce the cyclic decay rate of the Li–S batteries at 1C to only 0.025% and deliver a rate capacity of 744 mA h g^−1^ at 5C. A working schematic of the Ce–ZrOF@CC intermediate layer is shown in Fig. 4e. When employed as the intermediate layer of Li–S batteries, Ce–ZrOF@CC provides sufficient active sites for effective capture of polysulfides and rapid transformation of LiPSs, which is expected to inhibit the shuttle effect and enhance the electrochemical performance of the battery.

In addition, MOFs are highly important catalysts for energy storage and energy conversion systems.^165^ Research finds that introducing electrocatalysts into the matrix or interlayer materials can promote the slow conversion process, thereby enhancing the utilization rate of sulfur and battery performance.^150^ In the chemistry of Li–S batteries, the redox reaction involves multi-step and multi-phase transformations of sulfur during discharge/charging (Fig. 4b). In the sulfur reduction reaction, S_8_ is reduced stepwise to long-chain LiPSs at a voltage higher than 2.1 V. This process provides 25% of the theoretical capacity (418 mA h g^−1^). In this process, the adsorption, activation and desorption of LiPS are the key factors governing the reaction kinetics. In the second stage, the conversion from Li_2_S_4_ to Li_2_S_2_ contributes an additional 25% of the theoretical capacity. Finally, the solid–solid conversion from Li_2_S_2_ to Li_2_S is a rate-determining step that delivers the remaining 50% theoretical capacity (839 mA h g^−1^), as shown in Fig. 4c and d. This reaction step is instructive for sulfur reduction in Li–S batteries.^149^ Currently, a variety of dual-metal MOF catalysts are employed in battery systems; for example, NiCu-MOF catalysts^166^ and NiFe-MOF catalysts.^167^

#### 4.1.3 Lithium–air batteries

In the pursuit of higher energy density, LOBs, also known as lithium air batteries, have garnered considerable attention due to their higher theoretical energy density (3500 W h kg^−1^).^168^ Owing to their excellent physicochemical and electrochemical properties, MOFs have promising prospects for their application in LOBs.^169^ Based on the electrolyte type, they can be divided into four groups: proton type, aprotic type, mixed type and all-solid type.^170^ When these batteries are discharged, the reversible lithium dissolution/precipitation reaction occurs at the anode. The oxygen reduction/oxidation of the reaction products occurs on the positive pole.^171,172^ MOF-derived materials have large pore volumes, good pore structures and well-distributed catalysts, which are conducive to the regulation of quality transportation, oxygen-related redox reactions and the deposition of reaction products (such as Li_2_O_2_).^121,129^ The overall reaction of the battery is shown in reactions (4) and (5):

4 Li + 1/2O_2_ → 1/2Li_2_O_2_: the battery is discharged

5 1/2Li_2_O_2_ → Li + 1/2O_2_: the battery is charged.

There remain key challenges for the development of LOBs, such as the design and synthesis of high-efficiency catalysts for Li_2_O_2_ decomposition, the selection of electrolytes, and the passivation of porous cathodes induced by reaction products.^171,173^ Using MOFs as the precursor and mSiO_2_ as the template, cobalt-encapsulated catalysts (Co, N-CNF) confined within porous nitrogen-doped carbon nano-frameworks have been prepared. These catalysts retain their intrinsic structural features after MOF pyrolysis, inhibit the agglomeration of metal particles, and fully expose the catalytically active sites, thus enabling efficient catalytic decomposition of Li_2_O_2_. They effectively enhance the specific capacity of the battery (5288 mA h g^−1^) and the cycling stability (no obvious voltage decay after 500 cycles).^174^

Before LOBs can meet the requirements of actual applications, there are still numerous fundamental and technical challenges to be addressed, such as round-trip efficiency, energy density, and cycling durability. For the MOF materials, firstly, the deposition of reaction products and electrolyte decomposition byproducts may lead to blockage of the pores and active sites of the MOFs. Secondly, the active intermediates in the electrochemical process may induce structural damage to the MOFs. Finally, when used as a membrane, the functional active sites may be gradually occupied, leading to a decline in adsorption performance and thus a decrease in the electrochemical performance. In MOF-derived materials, the carbon component may be decomposed in the presence of Li_2_O_2_, resulting in poor cycling stability. Reducing surface defects and non-essential functional groups in the carbon matrix to enhance the structural stability may effectively mitigate the above issues and provide valuable insights for future research efforts. Gaining a better understanding of the basic chemical properties of LOB systems is of great significance for guiding the design and manufacture of effective MOF-derived materials.^121^

### 4.2. Application of COFs in lithium–sulfur batteries

**Fig. 5:**
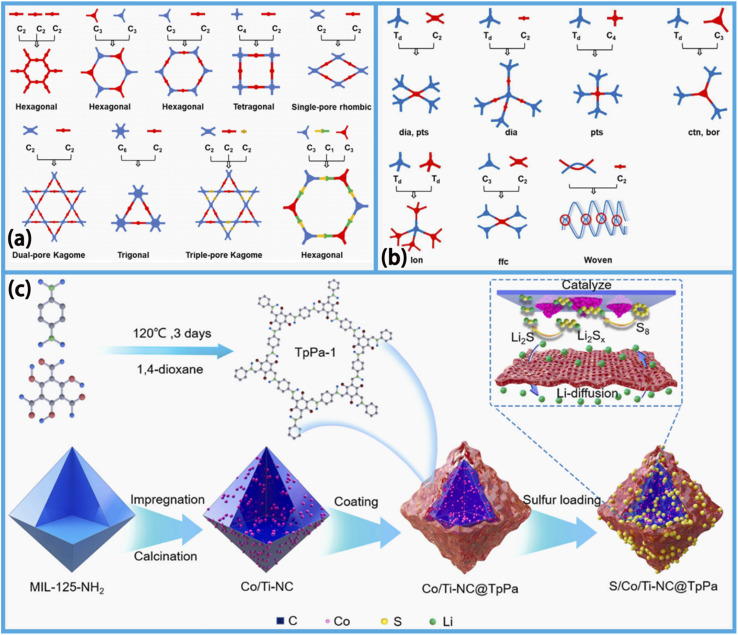
Basic topology of the COF design and constructed COFs: (a) 2D COFs and (b) 3D COFs, reproduced from ref. 175 with permission from Elsevier, copyright 2021. (c) Schematic diagram of the S/Co/Ti-NC@TpPa composite materials, reproduced from ref. 179 with permission from Elsevier, copyright 2023.

COFs are a new class of porous organic crystal materials that are synthesized from molecular building blocks containing light elements (such as C, H, O, N, or B atoms) *via* reticular chemistry. They achieve ordered covalent connectivity at the molecular level *via* periodic covalent bonding that can be further extended to form a porous framework structure in 2D or 3D space.^175,176^ Their unique properties, such as high porosity, diverse structural configurations, facile surface modifiability, large SSA, and good physical, chemical and thermal stability, have driven the vigorous development of COFs in the field of Li–S batteries.^175,177^ The excellent structural controllability of COFs affords critical support for tuning their physicochemical properties. Research shows that by choosing the appropriate connection motifs and building blocks, the backbone structures, pore sizes and pore geometry of COFs can be precisely designed.^178^ As seen in Fig. 5a and b, a variety of methods have been developed using distinct building units that allow the fabrication of COFs with different topological structures. By the rational and independent customization of the pore structure and functional groups of COFs, well-defined active sites can be introduced on the large, permanent and accessible surfaces to promote or catalyze the progress of target electrochemical reactions, providing a foundation for the rapid development of COFs in the field of electrode materials.^175^

Traditional Li–S batteries have some inherent drawbacks that need to be addressed, including the poor conductivity of sulfur and Li_2_S intermediates, sulfur volume expansion, the shuttle effect of the dissolution of polysulfide lithium intermediates on the electrolyte, and the electrochemical response issues during the slow reaction process of the electrolyte.^180–185^ As a typical type of porous material, COFs have been shown to effectively alleviate the performance bottlenecks of the aforementioned Li–S batteries.^178^ Theoretically, COFs containing multiple flexible units exhibit better adaptability to the dynamic behavior of active sulfur in batteries. Compared with Li–S batteries based on traditional conductive materials, the permanent 1D channels of 2D COFs not only provide diffusion channels for the electrolyte and Li^+^, but also selectively block the shuttle effect of polysulfides.^35^

To understand the mechanism by which COFs mitigate this shuttle effect, it is essential to consider the multi-step conversion reactions of sulfur in Li–S batteries. The discharge process involves the reduction of solid S_8_ to soluble long-chain lithium polysulfides (Li_2_S_*n*_, where 4 ≤ *n* ≤ 8), and ultimately to insoluble Li_2_S_2_/Li_2_S. The key steps related to the shuttle effect can be summarized as follows:

Overall reaction:

6 S_8_ + 16Li^+^ + 16e^−^ → 8Li_2_S

and Shuttle effect:

7 Li_2_S_*n*_(soluble) ⇌ Li_2_S_*n*−1_ + S + 2Li^+^ + 2e^−^ (*n* ≥ 4).

In one study, Ge *et al.* employed large, flexible monomers (TPT-CHO) as nodes to synthesize two flexible COFs containing triazine ring organic macromolecules (semi-rigid/flexible COFs-TPT(OH) and flexible COFs-TPT) as the cathode host materials for Li–S batteries. The strong coordination between –OH and O atoms and Li^+^ significantly inhibits the shuttle effect of polysulfides, and the highly efficient catalytic performance of COFs-TPT(OH)@S ensures no residual polysulfides remain at the end of discharge. Electrochemical tests show that COFs-TPT(OH)@S has the highest lithium-ion diffusion coefficient (7.77 × 10^−7^ cm^2^ s^−1^) and the smallest charge transfer impedance (6.7 Ω), and the reversible capacity after 1000 cycles at 0.5C is 732 mA h g^−1^, with a per-cycle decay rate of only 0.045%.^180^ In addition, the structural diversity of the COF building units provides abundant sites for the introduction of polar functional groups, thus enhancing the suppression of the shuttle effect.^35^ For instance, Zhang *et al.* introduced F functional groups into COFs to fabricate the 4F-COFs/PP (polypropylene) composite separator. Negatively charged channels induced by the high electronegativity of F can effectively suppress the shuttling of polysulfide anions by generating electrostatic repulsion. Meanwhile, the ordered porous structure and the superior electrolyte wettability boost the lithium-ion migration efficiency, allowing the durability to exceed 2000 h at 1 mA cm^−2^.^186^

However, the application of COFs in Li–S batteries is hindered by their intrinsically low electrical conductivity and the scarcity of active sites capable of catalyzing polysulfide redox reactions.^187^ Lin *et al.* proposed incorporating transition-metal-based porous carbon (PC) composites derived from MOFs into COFs. This advanced design strategy for the multifunctional core–shell sulfur cathode has an important impact on improving the polysulfide shuttle problem in sulfur batteries. This approach leverages the carbon/metal core inside the composite for sulfur sequestration and functions as a dedicated catalyst to accelerate the redox kinetics of sulfur species. In experimental tests, the S/Co/TiNC@TpPa battery (Fig. 5c) constructed from the core–shell structured Co/Ti-NC@TpPA composite shows excellent performance. In the experiments, the initial rate of the battery is as high as 1135 mA h g^−1^ at 0.2C, and after 500 cycles at 1C, the capacity decay rate per cycle is 0.05%. With a high sulfur load of 4.46 mg cm^−2^, the area capacity of the battery reaches 3.6 mA h cm^−2^.^179^

In energy storage battery applications, the porous structures and functionalized designs of MOFs and COFs are used to adapt to specific energy storage mechanisms. Both share the common characteristics of high specific surface area and controllable channels, providing ion adsorption/intrusion sites, shortening the ion transmission path, and allowing the energy storage performance to be optimized through modification of the active sites. However, MOFs achieve energy storage through redox reactions of metal ions/clusters or the double-layer energy storage of derived materials, with the metal active sites determining the redox capacity. COFs rely on ion adsorption and transmission as well as redox reactions of functional groups, and the regularity of pore channel determines the ion migration rate. Additionally, in terms of material performance requirements, it is necessary to ensure that the electronic/ion conductivity is suitable for charge transmission, chemical/electrochemical/thermal stability is sufficient to ensure cycle life, and the pore size/specific surface area/pore volume is adapted to the common rules for ion migration and storage.

## 5. Applications of supercapacitors

As the global economy grows, the dwindling supplies of non-renewable fossil fuels such as petrol and diesel, as well as the environmental concerns associated with their use, continue to spur researchers to explore new energy storage systems.^188,189^ The growing need for reliable energy has led to a fervent search for cutting-edge materials that are designed to further change the way energy is stored.^190^ In the past, humans predominantly relied on non-renewable resources (fossil energy) to generate the secondary energy (electricity) necessary for modern societal development.^191^ Hence, there is a pressing need to develop electrical energy storage systems with higher energy and power densities in order to meet the growing energy demand and mitigate greenhouse gas emissions.^192^ With the continuous advancement of modern science and technology, renewable energy sources such as wind power, nuclear power, and hydropower are gradually replacing fossil fuels for electricity generation.^193^ Electricity generation is becoming increasingly environmentally friendly, with a diminished environmental impact. The transition toward electricity replacing fossil fuels is becoming an inevitable trend of the era.^194^ Therefore, there is an increasingly urgent demand for eco-friendly and high-power energy storage systems.^195^ SCs have garnered considerable attention from researchers due to their fast charging and discharging rates, high power density, and excellent rate performance.^196^

SCs, also known as electrochemical capacitors, are a new type of energy storage device that exhibits characteristics between those of traditional capacitors and batteries, with unique properties distinct from traditional chemical power supplies.^197,198^ These advantages, including high power density, long cycle life and low maintenance costs, enable SCs to exhibit outstanding performance among numerous energy storage systems.^199–201^ They have a wide range of applications, including smart electronic devices, portable electronic devices, automotive systems, energy grids and defense sectors.^202^ There are three primary types of SCs: hybrid SCs, pseudocapacitors, and electric double-layer capacitors (EDLCs).^203,204^ The energy storage mechanisms of these three types of SCs are distinct, and can be represented by the following typical electrode reactions.

Electric double-layer capacitors (EDLCs), typically employing materials such as activated carbon in alkaline electrolytes (such as KOH), store charge *via* electrostatic adsorption/desorption of ions at the electrode–electrolyte interface without faradaic charge transfer.

8 C + OH^−^ ⇌ C|OH^−^ (positive electrode)

and

9 C + K^+^ ⇌ C|K^+^ (negative electrode)

Pseudocapacitors (such as RuO_2_ and MnO_2_):

Pseudocapacitors, exemplified by metal oxides like RuO_2_ and MnO_2_, rely on fast and reversible surface or near-surface faradaic redox reactions.

For RuO_2_ in acidic electrolyte:

10 RuO_2_ + *x*H^+^ + *x*e^−^ ⇌ RuO_2−*x*_(OH)_*x*_

and for MnO_2_ in neutral/alkaline electrolyte:

11 MnO_2_ + Li^+^ + e^−^ ⇌ LiMnO_2_.

Hybrid SCs combine capacitive and battery-like charge storage mechanisms, often integrating a battery-type electrode with a capacitive electrode. A representative example is the lithium-ion capacitor (LIC), which couples a pre-lithiated graphite anode with an activated carbon cathode.

12 Li_*x*_C_6_ ⇌ C_6_ + *x*Li^+^ + *x*e^−^ (battery-type anode)

13 C + PF_6_^−^ ⇌ C|PF_6_^−^ (EDLC-type cathode)

MOFs and COFs can be engineered to enhance these mechanisms by providing a high surface area for EDLC, introducing redox-active sites for pseudocapacitance, or serving as precursors for composite materials in hybrid systems.

In EDLCs, charge storage occurs through the electrostatic adsorption/desorption process at the electrode–electrolyte interface (EEI). The electrode surface undergoes a fast and reversible non-faradaic electrostatic adsorption process, resulting in high power density and low energy density.^205^ Hybrid SCs store charge through rapid and reversible faradaic reactions occurring at the electrode surface, which enables them to maintain high power density while also achieving higher energy density than EDLCs.^206^ Hybrid SCs are devices in which one electrode is an EDLC electrode, and the other is a battery electrode. Each of these electrodes utilizes different charge storage mechanisms: EDLC-type electrostatic adsorption and faradaic redox reactions. Pseudocapacitors exhibit excellent electrochemical performance, including high energy density, high energy, and outstanding cycling stability.^207^ Moreover, due to their dependence on rapid redox reactions occurring at or near the electrode surface region, pseudocapacitors typically possess higher charge storage capacity compared with EDLCs.^208^ With their excellent cycle life and power density, these three types of SCs play an important role in the modern market's ongoing quest for efficient, reliable and sustainable energy storage.^209^ However, due to the limitations of energy density, the further application of SCs has been significantly limited. The performance of SCs is closely related to their electrode materials. Therefore, it is of great significance to explore and develop new electrode materials to improve their electrochemical properties.^192^ At present, electrode materials used in SCs are generally divided into three categories, namely, transition metal oxides, carbon materials,^210–214^ and conductive polymers (CPs).^215–217^

In recent years, there has been a surge of interest in the fields of MOFs and COFs among researchers, with these novel materials emerging as pivotal components in enhancing SCs. This section aims to provide a comprehensive review of recent advancements in research and development of MOFs and COFs, and their derivatives, in the context of their application as electrode materials for SCs.

### 5.1. Applications of MOFs, MOF derivatives, and composite materials in SCs

MOFs are crystalline materials with unique properties, featuring a vast surface area conducive to molecular diffusion, precisely tunable and controllable porosity, and abundant active sites.^218^ Derivatives of these materials retain the distinctive ordered structures and networked voids of MOFs, exhibiting high porosity, large specific surface area (SSA), and high energy storage capacity.^219,220^ MOFs and their derivatives offer novel approaches to overcoming the performance limitations of SC electrode materials.

#### 5.1.1 Transition metal MOFs

MOFs containing transition metal ions, such as Ni, Co, V, Fe, and Zn, have emerged as key electrode materials for enhancing SC performance due to their tunable porous structures, abundant redox active sites, and synergistic effects among the components. K. Xia *et al.*^221^ tuned the molar ratio of metal salt to ligand (R_m_ : L) to synthesize 2D coordination-unsaturated Ni-MOF hierarchical nanosheets. The internal electric field reduced the bandgap, thus enhancing conductivity. At 1 A g^−1^, the specific capacity reached 746C g^−1^, with a capacity retention of 89.7% after 10 000 cycles. A. H. Anwer *et al.*^222^ used an ultrasonic-assisted hydrothermal method to synthesize the bimetallic, bi-ligand Co–V-MOF, exhibiting a *C*_g_ of 1711.1 F g^−1^ at 1 A g^−1^ and a retention rate of 92.21% after 10 000 cycles.

Combining transition metal MOFs with other materials or derivatizing them into metal selenides and oxides further enhances performance. A ternary Zn–Ni–Co–Se alloy combined with Ni–Co-LDH etched from MOFs was used to form a hierarchical structure on rGO-Ni foam, exhibiting a specific capacity of 387.2 mA h g^−1^ at 1 A g^−1^ and an assembled HSC with an energy density of 80.3 W h kg^−1^;^223^ MOF-derived ZnCo_2_O_4_/ZnO flower-like structures achieve a specific capacity of 803 F g^−1^ at 1 A g^−1^, with 91.04% capacity retention after 10 000 HSCs cycles;^224^ MOF-derived CoFe_2_O_4_@NiMn_2_O_4_ composite electrodes reach an energy density of 90.3 W h kg^−1^ in HSCs;^225^ MOF-derived CoSe_2_-Ni_3_Se_4_ nanosheets and MXene form a heterostructure *via in situ* growth on the substrate. The synergistic effect of the high conductivity of MXene and the active sites of CoSe_2_-Ni_3_Se_4_ reduces the charge transfer resistance, significantly enhancing the electrochemical performance of the hybrid SCs.^226^ Furthermore, Ni-doped V-MOF nanosheet arrays grown *in situ* on Ni foam exhibit a specific capacity of 516.5C g^−1^ at 1 A g^−1^, retaining 85.6% capacity after 12 000 cycles.^227^ Thus, transition metal MOFs offer an effective pathway for high-performance SCs through structural and compositional tuning. Table 3 summarizes the key performance parameters of the aforementioned transition metal MOF composites for SC electrodes, enabling an intuitive comparison of their overall performance.

**Table 3:** Important parameters of the MOF composites for SC electrode materials

Materials	Synthetic method	Cyclic stability (%); cycles	Specific capacitance (F g^−1^); current density (A g^−1^)	Specific energy (W h kg^−1^)
Co–V-MOF	Ultrasonic-assisted *in situ* hydrothermal	92.21; 10 000	187.5; 5	70.65 (ref. 222)
ZNC-Se@NC-LDH@rGO-NF	Hydrothermal	92.4; 10 000	226; 1	80.3 (ref. 223)
Ni-MOFs	Hydrothermal	89.7; 10 000	∼830; 1	53.1 (ref. 221)
MXene@CoSe_2_/Ni_3_Se_4_	—	80; 5000	283; 1	90 (ref. 226)
ZCO/ZnO	Hydrothermal	91.04; 10 000	161; 1	50.41 (ref. 224)
VNi-MOF NSAs/NF	One-step solvothermal	85.6; 12 000	∼574.6; 1	43 (ref. 227)
CFO@NMO	Hydrothermal	88.4; 10 000	312.8; 0.5	90.3 (ref. 225)
Ni/Co-MOF-NPC	Solvothermal	98.8; 6000	681; 10	55.4 (ref. 228)
MOF525-NC1.35	Carbonization transformation	77; 10 000	200; 5	—^229^
NGCA	High-temperature carbonization activation	93; 5000	244; 1	17.08 (ref. 230)
Ni-MOF/rGO	Hydrothermal synthesis	Increases by 49.34; 25 000	435.25; 1	76.96 (ref. 231)
Ni-MOF/CNF	Hydrothermal synthesis	88; 5000	742.2; 1	58.43 (ref. 232)

#### 5.1.2 Bimetallic MOFs and their derivatives

**Fig. 6:**
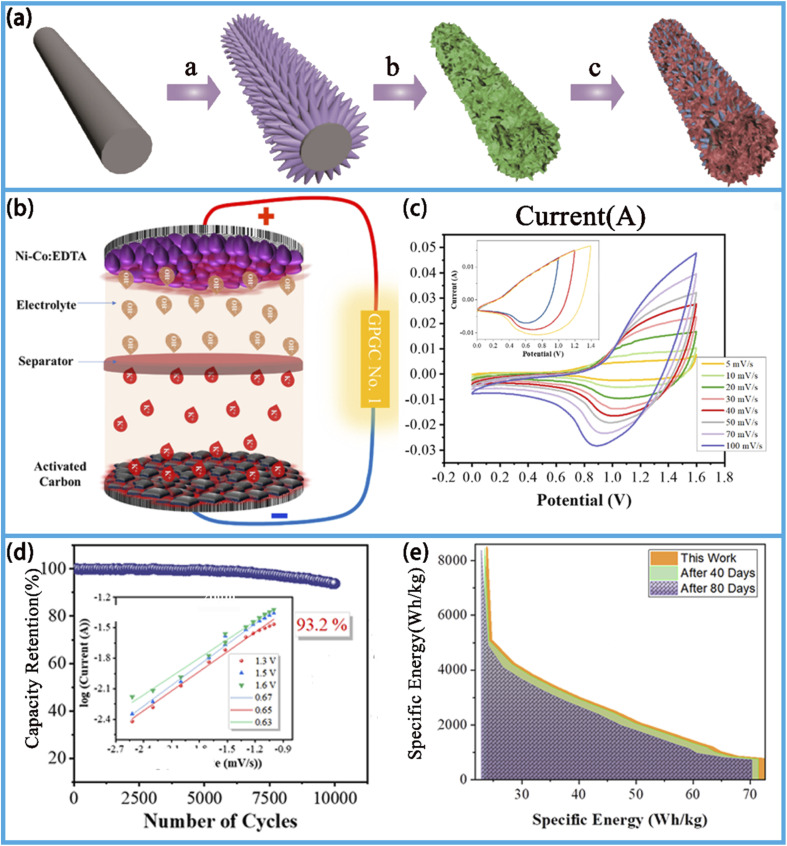
(a) Schematic of the preparation of CC/CoNi-MOF, reproduced from ref. 233 with permission from Elsevier, copyright 2020. (b) Power schematic of hybrid SCs. (c) CV analysis plots at different scan rates with different potential windows. (d) Capacity retention for 10 000 cycles. (e) Analysis of the power and energy after 40 and 80 days, reproduced from ref. 235 with permission from Elsevier, copyright 2024.

In the study of transition metal MOFs, bimetallic systems hold promise for further enhancing the performance of MOF-based energy storage devices. Xu *et al.* combined the layered structure of MOFs, characterized by fewer electrolyte ion diffusion pathways and shorter electron transfer channels, with the unique properties of carbon cloth (CC) to fabricate a SC cathode material. This material is composed of a carbon cloth (CC) substrate onto which bilayer CoNi-MOF nanosheets and nanotubes are assembled. The CC/CoNi-MOF nanosheets/nanotubes exhibit unique nanostructures and morphologies, yielding electrode materials with high specific surface area, high capacity and outstanding rate performance. Fig. 6a illustrates the synthesis of hierarchical CC/CoNi-MOF, featuring ultrathin nanosheets and nanotubes, using Co(OH)_2_ as a template. This hierarchical nanostructure significantly increases the contact surface area between the electrode material and the electrolyte, which is crucial for enhancing the electrochemical performance of the electrode. Experiments reveal that the hybrid SCs based on CC/CoNi-MOF and reduced graphene/CNTs exhibit a large areal capacitance of 846 mF cm^−2^ at 1 mA cm^−2^ (equivalent to 177.7 F g^−1^ at 0.21 g A g^−1^). This provides insights for the synthesis of other high-performance energy storage electrodes based on MOFs.^233^ Amidst tensions arising from energy crises and environmental concerns, researchers continue to explore novel materials to further enhance the performance of energy storage systems.^234^

J. Khan *et al.* published research in 2024 on synthesizing bimetallic MOFs using Ni and Co as transition metals. The researchers employed a hydrothermal synthesis method to prepare the Ni–Co MOF nanorods as the anode materials. By utilizing EDTA (ethylenediaminetetraacetic acid, an organic ligand with multiple advantages) as the organic ligand, the hydrothermal approach further enhanced the storage capacity and stability of the SC electrodes.^235^Fig. 6b illustrates the architecture of the Ni–Co-MOF device as the cathode component within the asymmetric structure of a hybrid SC, which facilitated its construction. The innovation of this approach lies in effectively enhancing the performance of the energy storage systems through structural optimization. To further evaluate the stability of this electrode material, the researchers conducted cyclic voltammetry tests, as shown in Fig. 6c. The cyclic voltammetry curves for this electrode assembly were obtained at scan rates ranging from 5 to 100 mV s^−1^, revealing symmetrical responses across varying scan rates. These data are crucial for subsequent verification of the stability of the device.

The cyclic potential evaluation of the experimental device was conducted by monitoring 10 000 consecutive charge–discharge cycles at a current density of 10 A g^−1^, as depicted in Fig. 6d. To validate the device's durability and reproducibility, the experimentally prepared hybrid structure was retested after being stored at room temperature for 40 days and 80 days. The experimental results are shown in Fig. 6e. As depicted, although the specific power and specific energy decrease after 40 and 80 days, the final stable electrochemical properties reach 71.43 W h kg^−1^ and 70.33 W h kg^−1^, respectively, demonstrating satisfactory performance.^235^

**Fig. 7:**
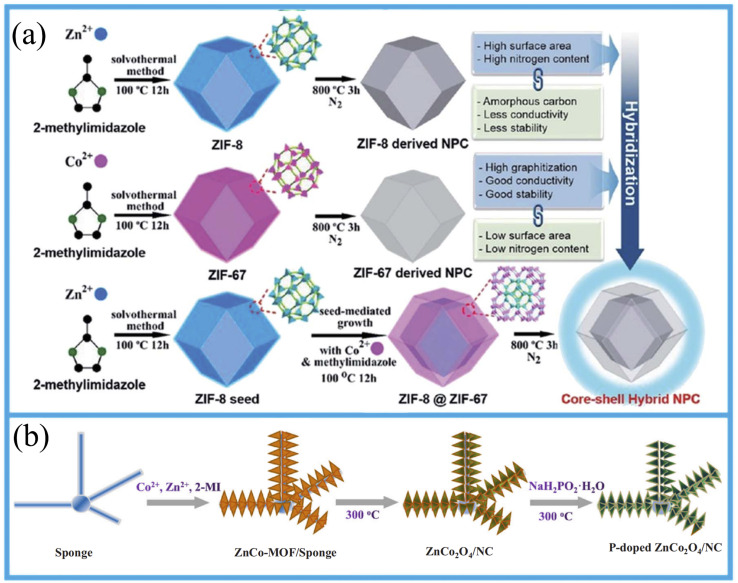
(a) Production of ZIF-8 and ZIF-67-derived nano-PC and their advantages and disadvantages. Reproduced with permission from the American Chemical Society and reproduced from ref. 239 with permission from the Royal Society of Chemistry, copyright 2020. (b) Schematic of the synthetic procedure used to prepare P-doped ZnCo_2_O_4_ on N-doped carbon, reproduced from ref. 238 with permission from Elsevier, copyright 2021.

Based on the principle that vacancies in nanostructured metal oxides significantly enhance the specific capacitance of electrode materials,^236,237^ Wei *et al.* conducted an in-depth exploration of MOF derivatives for developing novel electrode materials. They synthesized oxygen-rich vacancy P-doped ZnCo_2_O_4_ nanosheets (abbreviated as ZCP/NC) derived from nitrogen-doped carbon-loaded MOFs. They reported a method derived from the synthesis of ZnCo-MOFs to construct P-doped ZnCo_2_O_4_ nanosheets, with oxygen-rich vacancies coated on the surface of the nitrogen-doped carbon, as shown in Fig. 7b. Electrochemical testing results revealed that, under 1 A g^−1^ conditions, the maximum capacitance of the electrode material reached 1581.5 F g^−1^. Furthermore, the experimentally prepared composite material and activated carbon exhibit outstanding stability. When assembled as pseudocapacitors in asymmetric electrodes, their retention rate approached 90.6% after 5000 cycles.^238^

#### 5.1.3 Composites of MOFs with MXenes and CNTs

In recent years, MXene composites synthesized from MOFs have garnered significant attention due to their exceptional surface area and structural flexibility, sparking considerable interest in their use for the exploration of energy storage materials.^240^ Among these, the integration of MXenes with MOFs represents a new era of electrochemical synergy, effectively enhancing the performance of SCs by increasing the surface area and providing more active sites for electrochemical reactions.^241^ Despite the appeal of MOFs for energy-related applications, they still suffer from inherent challenges such as low conductivity and chemical sensitivity. These issues severely hinder their practical implementation and scalability. Recently, researchers discovered that the unique properties and structure of MXenes can potentially improve the conductivity and stability of pristine MOFs.^23^ First, the high conductivity of MXene frameworks enables the formation of efficient conductive pathways that effectively shorten ion diffusion routes. Furthermore, MXenes possess abundant negatively charged surface groups, making them excellent substrates for supporting MOF growth. This not only prevents the agglomeration of MXenes and MOF nanoparticles but also increases the accessible surface area. Additionally, strong interfacial interactions between MXene and MOFs significantly enhance the structural integrity and stability. Based on this, MXene@MOF composites hold great promise as functional materials for electrochemical applications. Furthermore, converting the MOFs into functional MOF-derived nanoparticles can optimize and enhance the electrochemical performance of MXene@MOF composites. It is noteworthy that the synergistic effects induced by the hybridization of MXene with MOFs or MOF-derived nanoparticles effectively address the inherent drawbacks of the individual components. Sani and his team investigated the preparation of MXene@MOF composites, particularly by utilizing MXene as a metal precursor for MOF synthesis. For the preparation of MOF/MXene composites, MXenes were added to a solution containing well-dissolved metal ions and organic ligands, with the composite formed by using the same synthesis methods used for pure MOFs. This approach includes room-temperature diffusion reactions, hydrothermal methods, and other technical approaches.^23^ The research conducted by K. J. *et al.* aimed to explore electrode materials suitable for hybrid supercapacitors with different charge storage mechanisms. They assembled carboxyl-functionalized graphene acid with amino-functionalized metal–organic framework UiO-66-NH_2_ through amide bonds to prepare the GA@UiO-66-NH_2_ hybrid material as the positive electrode, and Ti_3_C_2_T_*x*_ MXene as the negative electrode to construct an asymmetric supercapacitor. The energy density reached up to 73 W h kg^−1^, and the power density reached up to 16 kW kg^−1^. What is more remarkable is that, under a current density of 5.2 A g^−1^, this design could still maintain 88% of the initial capacitance after 10 000 cycles, and achieved a 100% Coulomb efficiency.^242^ These findings provide compelling evidence for the adoption of hybrid designs in fabricating more robust, high-power energy storage systems.

CNTs exhibit excellent electrical conductivity, large SSA, and high thermal stability, and they can be easily prepared at low cost. Given the precise properties required for SCs, CNTs have been extensively utilized in pseudocapacitors. Polypyrrole (PPy), polyaniline (PANI), and polythiophene (PTh) CNTs are commonly used in SC electrodes. Although these polymers offer relatively high theoretical specific capacitance values, their actual performance remains unsatisfactory. This is primarily due to the strong aggregation tendency of polymers, which causes a linear reduction in surface area. Consequently, the exposed surface area for interaction with the electrolyte decreases, leading to poor practical results. Research has revealed that composites of MOFs and CNTs can effectively overcome the inherent limitations of their parent materials. These composites eliminate the brittleness and agglomeration issues faced by CNTs while addressing the low conductivity problem associated with MOFs. TMOs derived from MOFs exhibit large SSAs and high conductivity. Since electrode materials undergo continuous expansion and contraction during SC charge–discharge cycles, the TMO-based electrodes typically exhibit poor cycling stability. This issue can be addressed by co-depositing mechanically stable CP (such as PANI) with TMO. He *et al.* designed the CuOx@mC@PANI@rGO composite, which was prepared *via* the *in situ* polymerization of aniline monomers. This composite exhibits an excellent *C*_g_ of 534.5 F g^−1^ at a current density of 1 A g^−1^.^243^

#### 5.1.4 A new type of MOFs: ZIF

The zeolite imidazole framework (ZIF) is a novel MOF material that has emerged in recent years. By combining organic and inorganic components, this approach enables the fabrication of materials with distinct particle shapes, exceptional porosity, and tailored surface functionality. As shown in Fig. 7a, ZIFs can be selectively pyrolyzed under controlled conditions to generate PC or metal compounds with unique nanostructures. ZIFs can also be synthesized *via* simple coordination chemistry of organic and inorganic components, allowing precise regulation and control of surface area, pore volume, and porous architecture. Consequently, the diverse and unique structures derived from them hold significant application potential as electrode materials for SCs. Among these, ZIF-67 and ZIF-8 have garnered attention from scientists as electrode materials for SCs.^239^ Yao *et al.* developed a promising strategy to overcome the conductivity barrier in MOF materials. They enhanced faradaic processes at the electrode interface by wrapping PANI chains around MOF crystals, with these PANI chains electrochemically deposited onto the MOF framework.^244^ MOF materials such as ZIF-67 and ZIF-8 have been successfully synthesized chemically. Compared with ZIF-67/POAP, SGO/ZIF-8/POAP, and pure graphene electrodes, the SGO/ZIF-67/POAP composite electrode exhibits a higher specific capacitance and maximum energy density. The SGO/ZIF-67/POAP composite electrode demonstrates superior capacitance values and maximum energy density relative to the ZIF-67/POAP, the SGO/ZIF-8/POAP, and the pure graphene electrodes. The *C*_g_ (specific capacitance) of the prepared electrodes is 825 and 670 F g^−1^, respectively. After 103 charge–discharge cycles on these two types of nanocomposite materials, it was found that they only lost 10% of their initial capacitance, which indicates extremely high electrochemical cycle stability. Furthermore, the energy densities of the SGO/ZIF-67/POAP and SGO/ZIF-8/POAP electrodes are 114.58 W h kg^−1^ and 111.77 W h kg^−1^, respectively. Compared with the electrochemical performance of more commonly used composites (such as MOFs and GO) at different current densities, the SGO/ZIF-67/POAP and SGO/ZIF-8/POAP composites exhibit superior performance due to their high conductivity.^245^ Furthermore, to validate the potential of the experimental materials as electrode materials for energy storage systems, researchers conducted additional electrochemical experiments. The ternary nanocomposite electrode (SGO/ZIF-67/POAP) exhibits a *C*_g_ of 825 F g^−1^ at a current density of 1 A g^−1^. Remarkably, the prepared electrode maintained 90% of its initial capacitance after 1000 cycles.

Notably, although the MOFs and their derivatives have demonstrated significant improvements in energy density and other aspects when used as container electrode materials for SCs, further research is still needed on composite materials formed by combining these frameworks with other high-performance materials. Evaluating the synthesis and experimental properties of such composites to elucidate how their chemical structures and architectures influence charge storage behavior will effectively advance the development of next-generation high-performance, green, and sustainable energy storage systems.

### 5.2. Application of COFs and their composite materials in SCs

COFs are crystalline organic porous materials formed by ordered covalent bonds between structural units.^246^ Their high porosity, tunable framework architectures, and charge-transfer-adaptive channels make COFs key materials for SC electrodes.^247^ Based on the geometric symmetry, COFs can be broadly classified into 2D and 3D structures.^248^ The functional group design not only adjusts the dimensional architectures of COFs but also directly governs their electrochemical performance. Therefore, the targeted incorporation of functional groups can endow COF materials with unique performance characteristics.

For SCs, 2D COFs (with porous channel characteristics that mimic the ion transport behavior of CNTs) facilitate electrolyte wetting of electrode surfaces and efficient ion adsorption, thereby enhancing specific capacitance.^249^ Specifically, the highly ordered porous structure of the COFs serves as an electron-conducting framework, providing an ideal ion-transport pathway at the EEI. Concurrently, their inherently large SSA offers an abundant number of ion-adsorption sites.^250^ These combined properties lay the foundation for enhanced SC performance.

To further validate the practical application potential of 2D COF-derived materials in SC electrodes, researchers have conducted in-depth studies *via* experimental approaches such as doping modification and structural optimization. Umezawa *et al.* provided key references for the preparation and performance evaluation of boron-doped COF-derived carbon materials. Umezawa *et al.* synthesized porous COFs with a C_9_H_4_BO_2_ structure (designated COF-5) *via* the direct boron carbide method. Thermogravimetric analysis confirms its excellent thermal stability, with the thermal decomposition temperature of COF-5 fluctuating only slightly around 600 °C. To obtain boron-doped PC materials, the team calcined COF-5 at 1000 °C. The resulting carbon material was washed with water under a nitrogen atmosphere to yield boron-doped PC (WCCOF-5). WCCOF-5 was further pulverized to prepare PCCOF-5 nanoparticles suitable for SC electrode films, and their electrochemical performance was evaluated to validate their energy storage potential. At a current density of 40 mA g^−1^, the pore characteristics and specific capacitance of PCCOF-5 and two commercial carbon materials, YP50F and MSP20, were tested, and the results show *C*_g_ values of approximately 82.9 F g^−1^, 99.6 F g^−1^, and 159.2 F g^−1^ for the three materials, respectively. Notably, despite the significantly lower SSA of PCCOF-5 (approximately 689 m^2^ g^−1^) compared with YP50F (1670 m^2^ g^−1^), its *C*_g_ (82.9 F g^−1^) was comparable with that of YP50F (99.6 F g^−1^). This suggests that PCCOF-5 possesses an optimized pore structure better suited for electrolyte ion transport. Furthermore, at a current density of 40 mA cm^−2^, PCCOF-5 exhibits the highest *C*_s_ among the three materials, at 15.3 µF cm^−2^.^251^

Currently, research is ongoing to identify highly efficient electrode materials for SCs and to synthesize materials with superior electrochemical performance. Carbon nanomaterials have emerged as representative electrode materials for SCs due to their high structural plasticity, large SSA, and excellent electrical conductivity. Furthermore, doping with heteroatoms such as boron, phosphorus, and sulfur can significantly enhance the properties of carbon nanomaterials. For instance, doping with heteroatoms such as carbon and nitrogen not only improves conductivity and capacitance but also enhances material wettability, outperforming pure carbon materials. Consequently, a key research direction involves deepening our understanding of the properties of existing energy storage material properties, overcoming the limitations of single materials, and constructing more efficient electrode material systems through rational combination strategies.

COFs, as suitable precursors for preparing carbon-based nanocomposite electrode materials for SCs, provide an effective pathway in this direction. Mewat *et al.* reported a COF-based composite system for SCs: triazine-based COF/GO nanocomposites were synthesized *via* both *ex situ* and *in situ* one-pot strategies. These composites were further converted into nitrogen-doped carbon (N-doped C)/reduced graphene oxide (rGO) composites through a simple carbonization step. Performance testing revealed that devices using the *in situ* prepared N-doped C/rGO (N-doped C/rGO_In) as the electrode material achieved a specific power of up to 400 W kg^−1^, a specific energy of 14.6 W h kg^−1^, and a capacitance performance degradation of only 14% after 3500 cycles. To systematically validate material performance, the researchers evaluated COF/GO, pure COF, N-doped C, and N-doped C/rGO as the SC electrode materials. Electrochemical impedance spectroscopy (EIS) was used to assess the conductivity and characterize the electrochemical performance, confirming that carbonized COF/rGO composites exhibit optimal capacitive properties.^252^

To conclude, COFs have evolved into key precursors for SC electrode materials, owing to their tailorable framework structures, superior porosity, and derived functional properties. Through the carbonization modification of single-component COFs or their composites with carbon-based materials (such as GO), the energy density, power density, and cycling stability of devices can be significantly enhanced, providing key technological support for the development of high-performance energy storage devices.

MOFs and COFs, as two prominent classes of porous framework materials, exhibit notable similarities in their applications in supercapacitors. Regarding the energy storage mechanism, both leverage their high specific surface area and ordered pore channels to enable efficient electrical double-layer capacitance. Additionally, their specific capacitance can be enhanced through pseudocapacitance, which is further facilitated by the introduction of redox-active sites. In terms of essential material properties, both require high electrical conductivity, robust structural stability, and well-designed micro/mesoporous architectures. Common performance optimization strategies include heteroatom doping, compositing with conductive matrices, and transformation into carbonized derivatives, which synergistically improve electrical conductivity, active site density, and structural integrity. Despite their significant potential for enhancing energy density, both MOFs and COFs face shared challenges, such as scalable synthesis, long-term cycling stability, and electrode–electrolyte interface optimization. Future research should focus on integrating their complementary structural and performance advantages through rational design and composite engineering to advance the development of next-generation high-performance energy storage systems.

## 6. Conclusions and prospects

### 6.1. Conclusion

With the continuous progress of society and the rapid development of science and technology, the associated industries, as the economic lifeline of a country, have naturally developed rapidly. However, this progression is followed by energy consumption, environmental pollution and other problems, which pose a great threat to human living environments. For more than two decades, researchers have been committed to exploring high-performance, versatile materials. The rapid development of advanced MOF/COF materials has triggered great interest, advances and breakthroughs in multifunctional applications. This is mainly due to their high SSA, active organic functional groups, and good pore structure and stability, which endow them with superior physical, chemical and biological properties. This review summarizes the synthesis and chemical structures of MOFs/COFs, as well as the latest progress in typical fields such as batteries and SCs, and highlights numerous important discoveries.

In terms of synthetic methods, solvothermal and hydrothermal syntheses feature mild reaction conditions, allowing precise control of crystal growth, and the products are formed with high crystallinity. Microwave-assisted synthesis can drastically reduce reaction times through the application of rapid and uniform heating, enhance the nucleation and growth kinetics of crystals, and offer a viable route for the efficient fabrication of high-performance materials. Mechanochemical synthesis does not require the use of solvents and can be solvent-free, rendering it more environmentally friendly, and it can also be used for efficient compounding with other materials. It has broad prospects in the preparation of low-cost materials. Ultrasonic chemical synthesis utilizes the ultrasonic cavitation effect to prepare nanoscale materials and synthesize special structures. In conclusion, each type of synthetic method has its own characteristics. For practical applications, the optimal synthetic method should be selected based on specific requirements of the application, such as research or production.

At the chemical structure level, the unique structures of MOFs and COFs are the core support for achieving high-performance energy storage. MOFs form 3D porous structures through the coordination of metal ions/clusters with organic ligands. Their regular pore systems provide an efficient path for ion transport, and the abundant metal active sites can directly participate in electrochemical reactions, significantly enhancing the energy storage capacity. COFs form 2D or 3D crystal porous structures through covalent bonds of light elements, featuring high stability, large SSA and uniform pore channels. These features not only increase the contact area between the electrode and the electrolyte, but also promote rapid ion diffusion, providing a structural guarantee for the optimization of energy storage performance.

In the field of batteries, these types of crystalline porous materials, with their ordered porous frameworks, can achieve rapid ion transport and storage without significant volume changes, providing a new solution to the problems of low power density and poor cycle stability of traditional batteries. MOFs exhibit excellent electrochemical efficacy in lithium-ion batteries, Li–S batteries and LOBs. Materials derived from MOFs can effectively mitigate the polysulfide shuttle effect of Li–S batteries and enhance the catalytic activity of LOBs through structural optimization. COFs, through the precise design of skeleton structure and functional groups, perform outstandingly in inhibiting polysulfide shuttling and optimizing ion transport kinetics, providing a new path for the development of high-energy-density batteries.

Within the realm of SCs, MOFs and their derivatives can substantially boost the specific capacitance and cycling stability of electrodes by regulating the composition of transition metals and allowing the fabrication of composite structures (such as combining with MXene and CNTs). The porous channel characteristics of COFs can simulate the ion transport function of CNTs, promoting electrolyte wetting and ion adsorption. Their highly ordered porous structure not only provides a framework for electron transport but also offers abundant ion adsorption sites, effectively enhancing the specific capacitance and energy density of SCs.

In conclusion, MOFs and COFs, with their unique advantages such as high SSA, tailorable pore architectures, abundant active sites and structural designability, have irreplaceable application value in the field of energy storage.

### 6.2. Challenges and future prospects

#### 6.2.1 Challenges

Although MOF/COF materials demonstrate great potential in numerous application fields, there are still some key issues to be addressed to further deepen our understanding of these materials and expand their application prospects. These problems include the following.

The bottleneck in the fabrication protocols makes it difficult for MOF/COF materials to balance large-scale production and economic efficiency. The commonly employed solvothermal and hydrothermal synthesis methods in the laboratory have long reaction cycles, high solvent consumption, and rely on high-pressure apparatus. When microwave-assisted synthesis is scaled up, it faces the problem of uneven heating, which leads to significant differences in product performance. Mechanochemical synthesis struggles to precisely control the particle size distribution and pore structure of the products, while sonochemical synthesis is limited by the reaction scale. Neither approach can directly meet the demands of industrial production. Meanwhile, the rare metal salts required by MOFs and the special organic ligands of COFs are expensive, and the synthesis cost is significantly higher than that of traditional carbon materials. Some synthetic solvents are also toxic or scarce, further increasing the cost of environmental remediation and the risk of interrupted raw material supply.

The insufficient adaptability to practical applications makes it difficult for MOF/COF materials to meet the actual service requirements of energy storage devices. Their porous structure is prone to adsorb solvent molecules in the electrolyte, can change the composition of the electrolyte in the channels, and can increase the resistance of ion diffusion. Furthermore, the material surface is prone to forming an unstable solid electrolyte interface (SEI) layer with the electrolyte. The metal sites of MOFs can also catalyze the repeated rupture and reconstruction of the film, exacerbating the increase in battery impedance. In terms of safety and thermal management, the high SSA endows the material with a robust adsorption capacity for electrolytes. At high temperatures, thermal runaway reactions are prone to occur. Some metal ions in MOFs may also catalyze the decomposition of the electrolyte to produce flammable substances, which limits their application in high-power scenarios. In addition, the MOFs/COFs in retired electrodes are mixed with various components such as binders and conductive agents, rendering their separation and purification difficult. Current recovery methods predominantly rely on strong acid dissolution, which not only leads to high energy consumption and serious pollution, but also, if the waste liquid and residue produced during the synthesis process are not properly treated, can cause heavy metal and organic pollution, which is in conflict with the environmental protection requirements for clean energy storage.

#### 6.2.2 Future prospects

In response to these challenges, relevant research has proposed numerous solutions, as follows.

Innovation in synthesis and fabrication processes: the synthetic process will develop in the direction of high efficiency, greenness and scalability. On the one hand, existing synthesis methods should be optimized by, for example, shortening the reaction cycle of solvothermal and hydrothermal synthesis through process integration and intelligent regulation of reaction parameters, improving the solvent recovery rate, and developing low-cost and environmentally friendly solvent systems to reduce the environmental footprint of the synthesis process. On the other hand, new synthetic technologies should be explored, such as continuous-flow synthesis and photo-assisted synthesis, to achieve batch preparation and performance homogenization of materials. In terms of cost control, low-cost precursors should be developed to replace rare metal salts and high-cost organic ligands, for instance, in the synthesis of covalent organic frameworks (COFs), low-cost organic ligands typified by tetrathiafulvalene (TTF) derivatives and aromatic amines derived from industrial by-products have demonstrated favorable application prospects. Compared with high-priced commercial organic ligands, these alternative ligands enable a 30–50% reduction in raw material costs, while retaining the exceptional structural regularity and functional properties inherent to COF materials.^253^ The application of industrial waste in material synthesis should also be explored to reduce raw material costs. Furthermore, ionothermal synthesis employs low-cost ionic liquids (ILs) as both solvents and templates. This approach enables the rapid fabrication of 3D COFs under ambient temperature and pressure, eliminating the need for expensive high-pressure reaction equipment and substantially reducing energy consumption.^254^ By optimizing the process to increase the synthetic yield, cost allocation under large-scale production can be achieved.

Application scenario expansion and system adaptation. In terms of application scenarios, MOF/COF materials will be deeply integrated in a diverse range of energy storage devices. In the field of batteries, dedicated MOF/COF-based electrode materials and intermediates will be developed to address the pain points of new types of batteries, such as Li–S batteries and LOBs, further enhancing the energy density and cycling stability of batteries. In the field of SCs, the focus will be on developing high-energy-density MOF/COF-based composite electrodes. By integrating them with CPs and transition metal oxides, the specific capacitance and cycling stability of the materials will be synergistically boosted. In terms of system adaptation, the collaborative design of materials and energy storage devices will be further advanced. In terms of interface optimization, the compatibility between the material and the electrolyte will be improved and the interface impedance will be reduced through surface modification, functional coating and other methods. In terms of thermal management, MOF/COF composite materials will be developed with both energy storage and heat conduction functions to enhance the thermal stability and safety of devices.

Interdisciplinary integration and advancement of fundamental research. In the future, the innovative development of the MOF/COF materials will be driven through interdisciplinary integration. By leveraging computational chemistry and artificial intelligence technologies, the structure–performance relationship of materials can be accurately predicted, guiding the design and synthesis of function-oriented MOFs/COFs and shortening the R&D cycle. By integrating advanced characterization techniques (such as *in situ* transmission electron microscopy and synchrotron radiation X-ray diffraction), the structural evolution, ion transport and reaction mechanisms of materials during the charge–discharge cycles are revealed in-depth, providing theoretical support for performance optimization. Strengthening of cross-disciplinary cooperation between materials science, electrochemistry, engineering and other fields should solve technical problems throughout the entire chain, such as material synthesis, device assembly and system integration, promoting the translation of MOF/COF materials from laboratory research to large-scale commercial applications, and providing key material support for alleviating the energy crisis and advancing sustainable energy development.

## Conflicts of interest

There are no conflicts of interest to declare.

## Data Availability

No primary research results, software or code have been included and no new data were generated or analysed as part of this review.
